# Influence of biochar and microorganism co-application on stabilization of cadmium (Cd) and improved maize growth in Cd-contaminated soil

**DOI:** 10.3389/fpls.2022.983830

**Published:** 2022-09-08

**Authors:** Fasih Ullah Haider, Muhammad Farooq, Muhammad Naveed, Sardar Alam Cheema, Noor ul Ain, Muhammad Arslan Salim, Cai Liqun, Adnan Mustafa

**Affiliations:** ^1^College of Resources and Environmental Sciences, Gansu Agricultural University, Lanzhou, China; ^2^Gansu Provincial Key Laboratory of Arid Land Crop Science, Gansu Agricultural University, Lanzhou, China; ^3^Department of Plant Sciences, College of Agricultural and Marine Sciences, Sultan Qaboos University, Seeb, Oman; ^4^Institute of Soil and Environmental Sciences, University of Agriculture, Faisalabad, Pakistan; ^5^Department of Agronomy, University of Agriculture, Faisalabad, Pakistan; ^6^Centre of Genomics and Biotechnology, Fujian Agriculture and Forestry University, Fuzhou, China; ^7^Faculty of Chemistry, Institute of Chemistry and Technology of Environmental Protection, Brno University of Technology, Brno, Czechia; ^8^Department of Agrochemistry, Soil Science, Microbiology and Plant Nutrition, Faculty of AgriSciences, Mendel University in Brno, Brno, Czechia; ^9^Institute for Environmental Studies, Faculty of Science, Charles University in Prague, Prague, Czechia

**Keywords:** cadmium toxicity, biochar, plant physiology, soil pollution, crop growth

## Abstract

Cadmium (Cd) is one the leading environmental contaminants. The Cd toxicity and its potential stabilization strategies have been investigated in the recent years. However, the combined effects of biochar and microorganisms on the adsorption of Cd and maize plant physiology, still remained unclear. Therefore, this experiment was conducted to evaluate the combined effects of biochar (BC) pyrolyzed from (maize-straw, cow-manure, and poultry-manure, and microorganisms [*Trichoderma harzianum* (fungus) and *Bacillus subtilis* (bacteria)], on plant nutrient uptake under various Cd-stress levels (0, 10, and 30 ppm). The highest level of Cd stress (30 ppm) caused the highest reduction in maize plant biomass, intercellular CO_2_, transpiration rate, water use efficiency, stomatal conductance, and photosynthesis rate as compared to control Cd_0_ (0 ppm). The sole application of BC and microorganisms significantly improved plant growth, intercellular CO_2_, transpiration rate, water use efficiency, stomatal conductance, and photosynthesis rate and caused a significant reduction in root and shoot Cd. However, the co-application of BC and microorganisms was more effective than the sole applications. In this regard, the highest improvement in plant growth and carbon assimilation, and highest reduction in root and shoot Cd was recorded from co-application of cow-manure and combined inoculation of *Trichoderma harzianum* (fungus) + *Bacillus subtilis* (bacteria) under Cd stress. However, due to the aging factor and biochar leaching alkalinity, the effectiveness of biochar in removing Cd may diminish over time, necessitating long-term experiments to improve understanding of biochar and microbial efficiency for specific bioremediation aims.

## Graphical Abstract

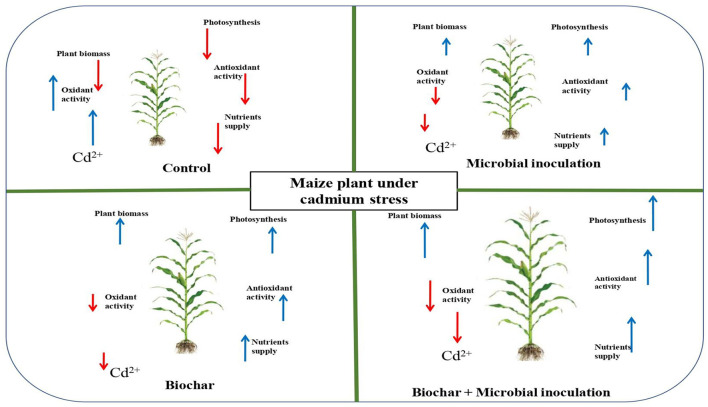

## Highlights

- Microbial inoculation improved the maize growth and reduced Cd bioavailability.
- Both microorganisms and biochar (BC) had synergistic effect to minimize Cd bioavailability.
- Co-application of microorganisms and BC improved antioxidant activity in plant under Cd stress.
- BC-induced reduction in Cd bioavailability was due to immobilization and adsorption.

## Introduction

Intensive industrialization has culminated in the gradual accumulation of cadmium (Cd) in arable soils (Zhang et al., 2019; Haider et al., 2021). Cadmium enters the environment through various anthropogenic emissions and activities (Abbas et al., 2017; Zeeshan et al., 2021). Owing to its higher mobility and bio-accumulation index in environments, the accumulation of Cd in plants grown in Cd-contaminated soils causes significant health issues for human beings and animals (Chen et al., 2016). Contamination of Cd affects various human body organs. It primarily accumulates in the kidneys and induces significant damage, i.e., kidney stones, pulmonary emphysema, and renal tubular damage (Mahajan and Kaushal, 2018). Cadmium replaces calcium (Ca) in minerals, because of having a similar chemical behavior, charges, and ionic radius (Gallego et al., 2012), and can be readily transported to the human body and deposited at a high level in various organs causing significant damages (Chen et al., 2016; Mahajan and Kaushal, 2018). Slow growth and chlorosis are common visual symptoms in plants produced by Cd-contamination (Uzoma et al., 2011; Farooq et al., 2020). Cadmium contamination of restricts the nutrients uptake, inhibits the carbon dioxide fixation, causes chlorophyll destruction resulting in reduction in net rate of photosynthesis and overall plant productivity (Gallego et al., 2012; Haider et al., 2021). Cadmium toxicity results in over-production of reactive oxygen species (ROS) causing damages to biological membranes and other sub-cellular organelles Zhao et al., 2013; Haider et al., 2022a; Zulfiqar et al., 2022. Therefore, developing effective strategies to mitigate the adverse effects of Cd on the ecosystem and soil rhizosphere is one of the major challenges. In this regard, a range of options are available (Abbas et al., 2017; Qin et al., 2020).

The application of biochar to for Cd stabilization in Cd-polluted soils has gained much attention in recent years (Shaaban et al., 2018). Biochar is characterized as an intrinsic porous-carbonaceous substance produced by pyrolysis of crop residues and organic manures (Bashir et al., 2017; Farooq et al., 2020). The application of biochar as an amendment in polluted soils causes significant reduction in the bioavailability and accumulation of Cd in horticultural and agronomic crops (El-Naggar et al., 2020). Several factors including feedstock type, pyrolysis temperature, retention time, and the physicochemical characteristics of biochar modulate the adsorption potential of Cd in polluted soils (Abbas et al., 2018; Yuan et al., 2018). The utilization of cow manure and poultry manure for soil enhancement in an unprocessed form can significantly contribute to certain environmental hazards and may cause food safety issues (Zhou et al., 2019). Untreated poultry and cow manures are major sources of ammonia volatilization which upon addition to the soil, may have adverse effects on plant growth (Uzoma et al., 2011). Poultry and cow manures contain fair amount of readily available nitrogen (N) and phosphorous (P). Its introduction into the soil will increase the chances of N and P runoff and may cause leaching losses, and surface and groundwater contamination (Zhang et al., 2018). The pyrolysis of poultry and cow manures into biochar can reduce the nutrient leaching, may reduce weight and volume, with improved physicochemical properties to promote plant growth (Uzoma et al., 2011; Zhou et al., 2019).

Several recent studies used microbial flora for the remediation of trace metal ions from contaminated soil (Hussain et al., 2021). Remediation of soil pollutants like trace metals by microorganisms (i.e., fungi, algae, and bacteria), is a cost-effective and eco-friendly remediation and stabilization strategy (Lata et al., 2019). In addition to remediation of trace metals from the soil, microbes can improve plant growth through better nutrient availability and update and supply of plant growth substances in metal contaminated soils (Ahmad et al., 2013). The microorganisms help remediation of trace metals through improved activity of 1-aminocyclopropane-1-carboxylate deaminase, redox transformation, and synthesis of exopolysaccharides and siderophores and biosurfactants (Nazli et al., 2020; Ejaz et al., 2021). The *Bacillus* species can help remediate the arsenic (As), mercury (Hg), nickel (Ni), chromium (Cr), lead (Pb), uranium (U), and Cd from polluted soils (Radhakrishnan et al., 2017). For instance, *Bacillus subtilis* inoculation of improved cereal plant growth through better water absorption and membrane stability in Cd-contaminated soil (Ahmad et al., 2014). Likewise, fungal species like *Trichoderma* spp., present in a wide range of soils, can help remediate trace metals and organic contaminants (Yaghoubian et al., 2019). For instance, *Trichoderma harzianum* inoculation in Cd-contaminated soil improved potato (*Solanum tuberosum*) tuber yield and reduced the Cd toxicity by 76% (Mohsenzadeh and Shahrokhi, 2014).

The integrated application of biochar and inoculated microbes may improve the soil organic carbon contents and enhance other soil physicochemical characteristics, which favors plant growth and productivity (Zhang et al., 2019). The co-application of biochar and microorganisms in trace metal polluted soils may help improving the stability of trace metals and soil alkalization, regulates the absorption of trace metals and promotes growth of plant species such as alfalfa (*Medicago sativa*) and soybean (*Glycine max*), (Zhang et al., 2019; Haider et al., 2021). However, the additive effect of combined biochar and microbe application in improving plant growth and soil properties is also associated with biochar application levels and their physicochemical characteristics (Yuan et al., 2018; Zhang et al., 2019). Nevertheless, after the soil application of biochar, the impact of microorganism inoculation on the less lethal effects of bioavailable contaminants like trace metals in the cereal cropping system remains skeptical. Moreover, in previous research (Haider et al., 2021), the interactive effects of different strains of microorganisms and biochar on soil characteristics, legumes crops (dicots with tap root system), and trace metal remediation from polluted soils are known, however, the effects on cereal crops including maize (monocot with fibrous root system) are still unknown. Therefore, this study was conducted to investigate the impact of microbial inoculation [*Trichoderma harzianum* (fungus) and *Bacillus subtilis* (bacteria)] and biochar application on soil physiochemical characteristics, maize (*Zea mays* L.) growth, and Cd stabilization and bioavailability in Cd-contaminated soil. This was hypothesized that the co-use of microbial inoculation and biochar application synergistically impact nutrients uptake, antioxidant activity, and maize growth by reducing metal uptake in plants cultivated in Cd-contaminated soil.

## Materials and methods

### Soil sampling and characterization

Farm soil was collected from Lijiabu town, Dingxi city, Gansu province, China (35°28′N, 104°44′E). The experimental soil belonged to Loessial soil (Chinese Soil Taxonomy Cooperative Research Group, 1995), which correlates to Calcaric Cambisols in WRB soil classification (FAO, 1990). The soil used was typical of cultivated soils in Northern China's Loess Plateau, mainly calcareous sandy loam soil having low organic matter and fertility. Soil samples were taken to determine physicochemical characteristics before the start of experiment. The collected soil samples were shade dried, ground, and sieving (sieve diameter 2 mm). The presence of trace metal ions in soil was affirmed by following the procedure mentioned by Rizwan et al. (2018). Briefly, dried soil (1 g) was taken in an Erlenmeyer flask, 10 mL nitric acid was added, and the flask was incubated overnight. The flask containing the mixture of soil and nitric acid was heated at 200°C and then was allowed to cool. After cooling, HNO_3_ (1 mL) and HClO_4_ (4 mL) were added to the flask containing solution and were heated at 200°C on a hot plate. After heating, the flask containing the solution was removed, when the HClO_4_ fumes come into sight, the mixture was allowed to cool. After cooling, 10 mL HCl was added to the mixture, and the flask was heated at 70°C for 1 h and was allowed to cool. After cooling, the final solution of the Erlenmeyer flask was taken into 50 mL having 1% HCl, and the solution was filtered using Whatman-filter paper #42. The soil pH was measured from soil-saturated paste with a pH meter (Satorious PB 10). The soil organic matter (OM) was estimated by the Walkly-Black method (Jackson, 1962). The soil used in the current study had a texture of sandy loam, free of carbonates, and alkaline nature. The experiment soil had 8.64 pH, 2.94 mS cm^−1^ electrical conductivity (EC), 6.40% organic matter, 3.71g kg^−1^ total C, 0.367g kg^−1^ total N, 0.777 g kg^−1^ total P, 0.605 mg kg^−1^ total Cd, 161.08 mg kg^−1^ available P, and 117.60 mg kg^−1^ available K.

### Preparation and characterization of biochar

To process biochar for the current study, the feedstock of poultry manure, maize straw, and cow manure were collected from an agricultural farm near Lanzhou, China. The maize straw was chopped. The maize straw and other feedstocks (poultry manure and cow manure) were air-dried and sieved by a 10-mesh-sieve. All feedstocks were pyrolyzed as detailed elsewhere (Zhou et al., 2019) using a stainless-steel furnace, with 550°C pyrolysis temperature, and having a 2 h retention time. The physicochemical properties of biochars were determined following Haider et al. (2021). The pH and EC of the biochars were measured using pH and EC meters, respectively, after shaking for 30 min in water with having water and biochar ratio 10:1 (Dume et al., 2015). Total nitrogen and carbon contents were determined using an elemental analyzer (Model IC 6000; Company Wayeal; Anhui Wanyl Science and Technology Cooperation Limited, Hefei, China), while potassium and phosphorous concentrations were determined using a flame photometer and spectrophotometer, respectively, after digesting biochar samples in the mixture of nitric acid/perchloric acid (di-acid). The physicochemical characteristics of biochars are given in Supplementary Table S1.

### Experimental details

The study was conducted in a greenhouse at Gansu Agricultural University, College of Resources and Environmental Sciences, Lanzhou, China from the 10 September 2020 to 25 November 2020. Dried farm soil (5 kg per pot) was added to each pot (27 cm × 20 cm × 24 cm). The experimental soil was spiked artificially with 0, 10 ppm (49.25 mg Cd(NO_3_)_2_ kg^−1^ soil), and 30 ppm (147.75 mg Cd(NO_3_)_2_ kg^−1^soil) as per treatment using cadmium nitrate as a Cd source before adding into the pots Inoculation culture of microorganisms i.e., *Trichoderma harzianum* (fungus) strain Y1 (MI_1_) (Liaquat et al., 2020), and *Bacillus subtilis* (bacteria) strain KC6 (MI_2_) (Xie et al., 2021), and combined microbial inoculation strains (MI_3_) were made in the lab of College of Resources and Environmental Sciences, Gansu Agricultural University, Lanzhou, China, in an Erlenmeyer flask, and then mixed with the artificially contaminated Cd soil by pouring the soil in pots on the plastic sheet, and allowed the microbes to get mixed properly and then again refilled the plastic pots with soils. The maize-straw biochar (BC_1_), cow-manure biochar (BC_2_), and poultry-manure biochar (BC_3_) were incorporated at 5% (w/w) in Cd contaminated soil and were properly mixed with microbes.

The experimental plan was comprised of 48 treatments in total, by following the completely randomized design with three replications. The treatments comprising soil with no artificial Cd contamination, no biochar, and no microorganism inoculation were classified as a control treatment (B_0_M_0_Cd_0_; Supplementary Table S2). Before sowing seeds in pots, maize seeds (Pioneer-30Y87), were sterilized with hydrogen peroxide (H_2_O_2_) having 10% (v/v) concentration for 20 min and washed thoroughly using distilled water. Six maize seeds were sown in each pot and were thinned 10 days after emergence to maintain three plants in each pot. During the experiment, every pot was fertilized with 0.34, 0.22, and 0.11g of N, P_2_O_5_, and K_2_O, respectively, using urea, di-ammonium phosphate, and sulfate of potash as a fertilizer source. The complete dose of K_2_O and P_2_O_5_ and the N half dose fertilizers were added to the soil before the sowing of maize in the pot and the remaining N dose was supplied to pots after 45 days of sowing by mixing the remaining quantity in water and applied uniformly to pots. Pots were irrigated, when and if needed, to ensure the optimal moisture level i.e., at the field capacity of the soil. Weeds were removed manually. The maize plants were harvested on 25 November 2020.

### Observations and measurements

#### Plant biomass

Harvested plants were further separated into leaves, roots, and shoots. The shoot and root of the plants were measured using a measuring tape. The plant fresh biomass was recorded immediately after harvest with a weighing balance, and the plant samples were put into an oven until constant weight to record dry biomass. To record the root dry weight of each plant, roots were collected from each pot, washed with distilled water, and put into the oven until constant weight to record root dry weight. The dried root and shoot samples were ground and used for determining the Cd concentration in respective plant parts.

#### Photosynthetic pigments and gas exchange parameters

Observations on gas exchange parameters were recorded 45 days after sowing (DAS) between 10:00 am and 11:00 am from fully developed penultimate leaves. The SPAD readings were recorded with a SPAD meter (Model SPAD 502, Company Konica Minolta, Osaka, Japan) (Haider et al., 2021). The photosynthesis activity and other gas exchange parameters including transpiration rate (Tr), intercellular CO_2_ (Ci), photosynthetic rate (Pn), water use efficiency (WUE) and stomatal conductance (Gs) were recorded with a portable infrared gas analyzer (Portable Gas Exchange Fluorescence System Model-GFS-3000, Company Walz Heinz GmbH, Eichenring, Effeltrich, Germany).

#### Determination of oxidative stress and antioxidants activity

The oxidative stress indicators i.e., electrolyte leakage (EL), hydrogen peroxide (H_2_O_2_), and malondialdehyde (MDA) were estimated from the fully mature middle maize leaves 60 DAS. The EL was determined by taking small pieces of maize leaves (0.25 g) in a tube containing 20 mL of distilled water. The initial EC was noted, and the tube was incubated at 100°C for 0.5 h then, the final EC of the solution present in the tube was noted. The EL of maize leaves was recorded as described by Dionisio-Sese and Tobita (1998).

The H_2_O_2_ and MDA contents in maize leaves were recorded according to the protocol of Zhang and Kirkham (Zhang and Kirkham, 1994) and Heath and Packer (Heath and Packer, 1968), respectively. For the determination of leaf MDA contents, thiobarbituric acid (0.1%, 5.0 mL) was added to a tube containing leaf samples (0.25 g), and the mixture was centrifuged at 4°C at 12,000 × *g* for 10 min. The supernatant was taken, and tricholroacetic acid (20%, 4.0 mL) and 0.5% thiobarbituric acid were added to the supernatant (1 mL). The mixture was heated for 30 min at 95°C. After cooling, the reaction mixture was centrifuged at 4°C at 10,000 × *g* for 10 min. After cooling, the absorbance of each sample (reaction mixture) was recorded at 450, 532, and 600 nm.

For the determination of leaf H_2_O_2_ contents, leaf samples (50 mg) were added into 50 mM phosphate buffer (3 mL) with pH 6.5. The reaction mixture was centrifuged at 6000 × *g* for 30 min at 4°C. After centrifugation, 1 mL of 0.1% Titanium sulfate (O_8_S_2_Ti) having H_2_SO_4_ (20%, v/v) was added to the solution mixture. The reaction mixture was centrifuged at 6,000 × *g* for 20 min at 4 °C. The absorption of the supernatant was observed at 410 nm wavelength.

The activities of antioxidant enzymes *viz*. peroxidase (POD), superoxide dismutase (SOD), and catalase (CAT) were recorded using a spectrophotometer (V-5800 visible spectrophotometer, Mesh Shanghai Yuanxi Instrument Co., Ltd., Shanghai, China). Maize leaf samples were digested in the phosphate buffer of 7.8 pH (0.05 M). The homogenized leaf samples were centrifuged at 12,000 × *g* at 4°C for 10 min. The activity of catalase (CAT) was recorded following the procedure mentioned by Aebi (1984). Briefly, enzyme extract (100 μL), H_2_O_2_ solution (300 mM, 100 L), and phosphate buffer (50 mM, 2.8 mL) was added to the assay mixture (3 mL). The CAT activity was recorded by recording the absorbance at 240 nm wavelength. The enzymatic activities of SOD and POD were documented according to the procedure mentioned by Zhang, (Zhang, 1992). All the reagents used were of analytical quality purchased and provided by the College of Resources and Environmental Sciences, Gansu Agricultural University, Lanzhou, China.

#### Cadmium in maize root and shoot, and rhizosphere

The maize root and shoot dried samples were properly digested into 10 mL solution containing perchloric acid and nitric acid (1:3 v/v), by placing samples overnight and then nitric acid (5 mL) was added following Ryan et al. (2001). The Cd concentration in maize shoots, roots, and soil was determined by atomic absorption spectrophotometer (Model 3200-C, S/N: KETC0478, Company Heinz Walz GmbH, 91090, Effecltrich, Germany).

#### Microscopic characterization of biochar

Biochar samples sieved with a mesh diameter of 2 mm were subjected to Electron microscopy imaging at Gansu Agricultural University in Lanzhou, China, using a Leo ® 1430VP Scanning Electron Microscope (ZEISS, Germany) according to procedure mentioned by Liu et al. (2020). Until imaging, the samples were taped to a stub of dual-sided carbon tape. The samples were then coated with a thin film of gold to make the base electrically conductive. The scanning electron images show the surface structure of the object. During the surface study, the beam conditions were 5.0 kV and 1.5 nA, having a spot size of 150.

### Statistical analysis

Experimental data were analyzed by analysis of variances (ANOVA) technique using statistical software SPSS (version 22.0; SPSS Inc., Chicago IL, USA). Mean values of treatments were compared *via* Duncan's new multiple range test at *P* ≤ 0.05. Microsoft Excel was used to compute Pearson's correlations between various traits. Graph-Pad was used to construct a graphical representation of the experimental results. The canonical correspondence analysis (CCA) was performed to show major gradients in the combination of response variables of data taken from independent and random samples. The experimental data were firstly subjected to Hellinger-transformation for the confirmation of linearity and normality of data. The impact of Cd and remediation strategies (microbes and biochar) on response variables was also tested by an ANOVA-like Monte Carlo permutation test. The CCA analysis was performed in an R-statistical package using the vegan library.

## Results

### Plant biomass

The root, shoot length, and overall biomass of maize significantly differed with the inoculation of microorganisms and incorporation of biochar under Cd-contaminated soil (Supplementary Table S3). Cadmium stress (30 ppm) reduced the root and shoot length and plant biomass by 18.78, 19.86, and 27.06%, respectively than the control. However, the application of biochar and microorganisms caused significant improvement in root length, shoot length, and plant biomass (Tables 1, 2). Application of cow manure biochar (BC_2_) without microbial inoculation (MI_0_), the shoot length and dry biomass of maize were significantly enhanced to 61.94 ± 2.34 cm and 14.27 ± 1.12 g compared to control i.e., 33.91 ± 2.95 cm and 7.52 ± 0.64 g, respectively. Among the microbial inoculation treatments, the highest shoot length and plant biomass were recorded from the combined inoculation of *Trichoderma* and *Bacillus* (MI_3_) that was 21.38 and 12.91%, higher than control (having no inoculant; MI_0_), respectively (Table 2).

**Table 1:** Effect of integrated application of biochar and microbes on root length, root fresh biomass, and root dry biomass of maize grown in cadmium (Cd)-contaminated soil.

**CD**	**MI**	**BC_0_**	**BC_1_**	**BC_2_**	**BC_3_**	**Mean (Cd × MI)**
Root length (cm)						
Cd_0_	MI_0_	33.91 ± 2.95 tuv	41.59 ± 1.75 klm	54.58 ± 2.24 b	48.87 ± 1.66 c-f	44.74 ± 8.97 B
	MI_1_	44.11 ± 3.73 h-k	37.71 ± 1.65 n-r	45.29 ± 2.52 g-j	47.47 ± 1.82 d-g	43.65 ± 4.19 BC
	MI_2_	36.14 ± 3.12 o-t	37.05 ± 0.99 n-t	49.82 ± 1.30 cde	48.83 ± 1.83 c-f	42.96 ± 7.37 BC
	MI_3_	47.04 ± 2.38 e-h	50.71 ± 2.20 c	61.94 ± 2.34 a	56.25 ± 1.93 b	53.99 ± 6.52 A
Cd_1_	MI_0_	36.30 ± 2.31 o-t	31.33 ± 2.16 vw	38.34 ± 2.35 n-q	36.80 ± 1.58 n-t	35.69 ± 3.04 DE
	MI_1_	39.91 ± 2.81 lmn	35.20 ± 2.26 q-u	41.60 ± 2.17 klm	50.68 ± 2.07 cd	41.85 ± 6.48 C
	MI_2_	43.81 ± 2.70 ijk	34.95 ± 1.06 r-u	49.47 ± 1.96 c-f	48.12 ± 2.02 c-g	44.09 ± 6.55 BC
	MI_3_	42.48 ± 2.59 jkl	38.14 ± 1.13 n-r	48.03 ± 1.55 c-g	48.49 ± 1.94 c-g	44.29 ± 4.92 BC
Cd_2_	MI_0_	33.92 ± 1.76 tuv	25.43 ± 2.02 x	35.57 ± 2.01 q-u	33.95 ± 1.90 tuv	32.22 ± 4.59 F
	MI_1_	32.56 ± 3.21 uv	34.40 ± 1.59 s-v	46.91 ± 2.49 e-i	38.84 ± 1.90 m-p	38.18 ± 6.39 D
	MI_2_	39.30 ± 1.90 l-o	28.85 ± 2.10 w	35.66 ± 1.87 p-u	34.49 ± 1.79 s-v	34.57 ± 4.33 EF
	MI_3_	50.30 ± 1.63 cd	37.59 ± 2.42 n-s	46.61 ± 0.73 f-i	47.78 ± 1.47 c-g	45.57 ± 5.54 B
Mean (BC)		39.98 ± 5.66 B	36.08 ± 6.38 C	46.15 ± 7.71 A	45.05 ± 7.15 A	
LSD (p 0.05) value	Cd × MI = 2.71; BC = 1.22; Cd × MI × BC = 6.64					
Root fresh biomass (g)						
Cd_0_	MI_0_	19.70 ± 1.20 t-w	22.80 ± 2.03 q-t	31.71 ± 2.56 c-f	29.61 ± 3.09 e-j	25.96 ± 5.65 BCD
	MI_1_	21.10 ± 1.05 s-v	23.15 ± 0.53 qrs	28.50 ± 2.95 f-k	26.40 ± 1.22 j-p	24.79 ± 3.30 DE
	MI_2_	19.30 ± 0.67 u-x	24.14 ± 3.01 n-s	28.85 ± 2.55 f-k	27.43 ± 2.75 h-m	24.93 ± 4.24 CDE
	MI_3_	22.41 ± 1.16 r-u	27.29 ± 1.51 h-n	41.14 ± 2.96 a	32.36 ± 0.69 b-e	30.80 ± 8.00 A
Cd_1_	MI_0_	16.49 ± 0.99 wxy	23.87 ± 0.92 o-s	35.29 ± 1.45 b	28.27 ± 1.10 g-m	25.98 ± 7.88 BCD
	MI_1_	15.18 ± 1.87 yz	23.04 ± 0.08 qrs	30.38 ± 3.04 d-h	28.33 ± 2.93 g-l	24.23 ± 6.78 E
	MI_2_	17.33 ± 0.56 wxy	25.11 ± 2.84 m-r	33.68 ± 1.30 bc	31.55 ± 1.88 c-f	26.92 ± 7.36 B
	MI_3_	17.37 ± 0.34 wxy	21.12 ± 1.58 s-v	35.40 ± 2.07 b	29.95 ± 1.35 d-i	25.96 ± 8.21 BCD
Cd_2_	MI_0_	12.16 ± 1.79 z	19.54 ± 2.49 uvw	31.40 ± 2.77 c-g	21.41 ± 2.68 stu	21.13 ± 7.93 F
	MI_1_	21.24 ± 2.67 stu	21.56 ± 2.70 stu	29.31 ± 2.96 e-j	26.85 ± 2.27 i-o	24.74 ± 3.99 DE
	MI_2_	17.97 ± 2.34 v-y	25.15 ± 2.58 l-r	33.76 ± 3.00 bc	28.99 ± 1.56 f-k	26.47 ± 6.67 BC
	MI_3_	16.12 ± 2.16 xy	23.38 ± 2.89 p-s	33.15 ± 2.03 bcd	26.01 ± 2.18 k-q	24.67 ± 7.04 DE
Mean (BC)		18.03 ± 2.91 D	23.35 ± 2.04 C	32.71 ± 3.56 A	28.10 ± 2.87 B	
LSD (0 0.05) value	Cd × MI = 1.61; BC = 0.93; Cd × MI × BC = 3.21					
Dry biomass (g)						
Cd_0_	MI_0_	7.52 ± 0.64 tuv	9.21 ± 0.79 k-q	11.99 ± 0.85 bcd	11.33 ± 0.97 def	10.01 ± 2.04 BCD
	MI_1_	8.45 ± 0.31 p-t	9.18 ± 0.13 k-q	10.62 ± 1.18 e-i	9.87 ± 0.49 h-m	9.53 ± 0.93 CDE
	MI_2_	8.15 ± 0.46 q-u	10.37 ± 0.69 f-j	11.32 ± 1.10 def	11.34 ± 0.72 def	10.30 ± 1.50 B
	MI_3_	8.45 ± 0.30 p-t	10.41 ± 0.58 f-j	14.27 ± 1.12 a	12.06 ± 0.35 bcd	11.30 ± 2.47 A
Cd_1_	MI_0_	6.25 ± 0.48 w	9.46 ± 0.47 j-p	12.68 ± 0.68 bc	10.43 ± 0.28 f-j	9.70 ± 2.67 CDE
	MI_1_	6.27 ± 0.54 w	8.86 ± 0.15 m-s	11.33 ± 1.12 def	10.86 ± 0.87 e-h	9.33 ± 2.31 EF
	MI_2_	7.28 ± 0.06 uvw	9.80 ± 0.88 h-n	11.65 ± 0.18 b-e	11.37 ± 0.59 def	10.03 ± 2.00 BC
	MI_3_	7.14 ± 0.13 uvw	8.68 ± 0.44 o-s	12.74 ± 0.48 b	11.07 ± 0.38 d-g	9.91 ± 2.49 BCD
Cd_2_	MI_0_	4.94 ± 0.73 x	7.83 ± 0.67 s-v	10.74 ± 1.00 e-h	7.85 ± 0.97 s-v	7.84 ± 2.37 H
	MI_1_	7.92 ± 1.02 stu	8.07 ± 0.97 r-u	10.28 ± 1.16 f-k	9.59 ± 0.88 i-o	8.97 ± 1.16 FG
	MI_2_	6.80 ± 0.75 vw	9.36 ± 0.84 j-p	11.61 ± 0.85 cde	10.11 ± 0.54 g-l	9.47 ± 2.01 DEF
	MI_3_	6.18 ± 0.81 w	8.69 ± 1.10 n-s	11.13 ± 0.78 d-g	9.06 ± 0.78 l-r	8.77 ± 2.03 G
Mean (BC)		7.11 ± 1.07 D	9.16 ± 0.80 C	11.70 ± 1.11 A	10.41 ± 1.18 B	
LSD (p 0.05) value	Cd × MI = 0.55; BC = 0.32; Cd × MI × BC = 1.11					

Values are representing means ± standard deviation (SD) of three replication. Means within a column sharing the same letters do not differ significantly different at P ≤ 0.05. BC = biochar types; Cd = artificial cadmium stress; MI = microbial inoculation; BC0 = zero biochar; BC1 = maize-straw synthesized biochar, BC2 = cow-manure synthesized biochar; BC3 = poultry-manure synthesized biochar; MI0 = no microbial inoculation; MI1 = Trichoderma harzianum (fungus) microbial inoculation; MI2 = Bacillus subtilis (bacteria) microbial inoculation; MI3 = combined Trichoderma harzianum and Bacillus subtilis microbial inoculation; Cd0 = 0 ppm Cd; Cd1 = 10 ppm Cd; Cd2 = 30 ppm Cd.

**Table 2:** Effect of integrated application of biochar and microbes on shoot length, shoot fresh, and dry biomass of maize grown in cadmium (Cd)-contaminated soil.

**CD**	**MI**	**BC_0_**	**BC_1_**	**BC_2_**	**BC_3_**	**Mean (Cd × MI)**
Shoot length (cm)						
Cd_0_	MI_0_	33.91 ± 2.95 s	59.42 ± 2.50 f	77.97 ± 3.20 b	69.82 ± 2.37 cd	60.28 ± 19.15 B
	MI_1_	44.11 ± 3.73 m-q	53.88 ± 2.36 ghi	64.71 ± 3.60 e	67.81 ± 2.60 cde	57.63 ± 10.81 CD
	MI_2_	36.14 ± 3.12 rs	52.93 ± 1.41 g-j	71.18 ± 1.85 cd	69.76 ± 2.61 cde	57.50 ± 16.48 CD
	MI_3_	47.04 ± 2.38 k-o	72.45 ± 3.14 c	88.49 ± 3.35 a	80.36 ± 3.74 b	72.08 ± 17.93 A
Cd_1_	MI_0_	36.30 ± 2.31 rs	44.76 ± 3.96 l-p	54.78 ± 3.36 fgh	52.57 ± 2.26 g-j	47.10 ± 8.39 F
	MI_1_	39.91 ± 2.81 pqr	50.62 ± 4.76 g-k	59.43 ± 3.10 f	72.40 ± 4.34 c	55.59 ± 13.76 D
	MI_2_	43.81 ± 3.59 n-q	49.92 ± 1.52 h-k	70.67 ± 2.80 cd	68.75 ± 2.89 cde	58.29 ± 13.44 BC
	MI_3_	42.48 ± 3.56 opq	54.49 ± 3.61 fgh	68.62 ± 3.85 cde	69.27 ± 2.78 cde	58.71 ± 12.79 BC
Cd_2_	MI_0_	33.92 ± 3.72 s	36.33 ± 3.88 rs	50.81 ± 2.88 g-k	48.50 ± 4.71 j-n	42.39 ± 8.50 G
	MI_1_	32.56 ± 3.21 s	49.15 ± 4.27 i-m	67.02 ± 5.54 de	55.49 ± 4.71 fg	51.05 ± 14.38 E
	MI_2_	39.30 ± 3.88 qr	41.21 ± 4.00 pqr	50.94 ± 4.67 g-k	49.27 ± 4.55 i-l	45.18 ± 5.78 F
	MI_3_	50.30 ± 4.63 h-k	53.70 ± 4.87 ghi	66.58 ± 3.83 de	68.26 ± 3.99 cde	59.71 ± 9.04 BC
Mean (BC)		39.98 ± 5.66 D	51.57 ± 9.11 C	65.93 ± 11.01 A	64.36 ± 10.21 B	
LSD (p 0.05) value	Cd × MI = 2.53; BC = 1.46; Cd × MI × BC = 5.07					
Fresh biomass (g)						
Cd_0_	MI_0_	23.95 ± 1.17 wxy	37.66 ± 2.03 cd	43.83 ± 0.76 a	38.02 ± 1.18 cd	35.87 ± 8.43 B
	MI_1_	30.69 ± 1.85 l-o	34.20 ± 0.96 fgh	34.62 ± 0.87 efg	36.33 ± 1.49 de	33.96 ± 2.37 C
	MI_2_	28.05 ± 1.11 p-t	34.52 ± 1.08 efg	37.46 ± 0.93 d	37.34 ± 0.77 d	34.34 ± 4.41 C
	MI_3_	27.58 ± 0.79 q-u	39.70 ± 1.48 bc	44.01 ± 1.18 a	41.04 ± 2.16 b	38.08 ± 7.23 A
Cd_1_	MI_0_	22.58 ± 0.71 yz	31.27 ± 1.49 k-n	34.00 ± 1.95 ghi	29.63 ± 0.80 n-q	29.37 ± 4.87 EF
	MI_1_	28.58 ± 0.96 o-s	28.96 ± 1.73 o-r	33.65 ± 1.02 g-j	38.21 ± 2.21 cd	32.35 ± 4.54 D
	MI_2_	29.76 ± 1.87 m-p	32.02 ± 0.60 i-l	34.47 ± 0.88 efg	33.81 ± 1.69 g-j	32.51 ± 2.11 D
	MI_3_	27.77 ± 1.12 p-u	30.27 ± 1.97 l-o	36.15 ± 1.39 def	37.29 ± 1.61 d	32.87 ± 4.58 D
Cd_2_	MI_0_	21.15 ± 1.27 z	22.02 ± 1.47 yz	25.76 ± 1.41 uvw	27.40 ± 1.59 r-u	24.08 ± 2.98 H
	MI_1_	22.90 ± 1.48 xyz	26.57 ± 1.48 s-v	33.28 ± 1.47 g-k	32.34 ± 1.41 h-l	28.77 ± 4.91 F
	MI_2_	24.79 ± 1.79 vwx	24.08 ± 1.77 wxy	26.63 ± 1.79 s-v	26.04 ± 1.79 t-w	25.38 ± 1.16 G
	MI_3_	27.52 ± 1.25 q-u	28.01 ± 1.86 p-t	32.02 ± 1.16 i-l	31.81 ± 1.39 j-m	29.84 ± 2.41 E
Mean (BC)		26.28 ± 3.08 C	30.77 ± 5.27 B	34.66 ± 5.52 A	34.10 ± 4.71 A	
LSD (p 0.05) value	Cd × MI = 1.06; BC = 0.61; Cd × MI × BC = 2.13					
Dry biomass (g)						
Cd_0_	MI_0_	2.85 ± 0.17 wxy	4.81 ± 0.29 cd	5.69 ± 0.11 a	4.86 ± 0.17 cd	4.55 ± 1.20 B
	MI_1_	3.81 ± 0.26 l-o	4.31 ± 0.14 e-h	4.37 ± 0.12 efg	4.62 ± 0.21 de	4.28 ± 0.34 C
	MI_2_	3.44 ± 0.16 p-t	4.36 ± 0.15 efg	4.78 ± 0.13 d	4.76 ± 0.11 d	4.33 ± 0.63 C
	MI_3_	3.37 ± 0.11 q-u	5.10 ± 0.21 bc	5.72 ± 0.17 a	5.29 ± 0.31 b	4.87 ± 1.03 A
Cd_1_	MI_0_	2.65 ± 0.10 yz	3.90 ± 0.21 k-n	4.29 ± 0.28 f-i	3.66 ± 0.11 n-q	3.62 ± 0.70 E
	MI_1_	3.51 ± 0.14 o-s	3.57 ± 0.25 o-r	4.24 ± 0.15 g-j	4.89 ± 0.32 cd	4.05 ± 0.65 D
	MI_2_	3.68 ± 0.27 m-p	4.00 ± 0.09 i-l	4.35 ± 0.13 efg	4.26 ± 0.24 g-j	4.07 ± 0.30 D
	MI_3_	3.40 ± 0.16 p-u	3.75 ± 0.28 l-o	4.59 ± 0.20 def	4.76 ± 0.23 d	4.12 ± 0.65 D
Cd_2_	MI_0_	2.45 ± 0.18 z	2.57 ± 0.21 yz	3.11 ± 0.20 uvw	3.34 ± 0.23 r-u	2.87 ± 0.43 G
	MI_1_	2.70 ± 0.21 xyz	3.22 ± 0.21 s-v	4.18 ± 0.21 g-k	4.05 ± 0.20 h-l	3.54 ± 0.70 E
	MI_2_	2.97 ± 0.26 vwx	2.87 ± 0.25 wxy	3.23 ± 0.26 s-v	3.15 ± 0.26 t-w	3.05 ± 0.17 F
	MI_3_	3.36 ± 0.18 q-u	3.43 ± 0.27 p-t	4.00 ± 0.17 i-l	3.97 ± 0.20 j-m	3.69 ± 0.34 E
Mean (BC)		3.18 ± 0.44 C	3.82 ± 0.75 B	4.38 ± 0.79 A	4.30 ± 0.67 A	
LSD (p 0.05) value	Cd × MI = 0.15; BC = 0.09; Cd × MI × BC = 0.30					

Values are representing means ± standard deviation (SD) of three replication. Means within a column sharing the same letters do not differ significantly different at P ≤ 0.05. BC = biochar types; Cd = artificial cadmium stress; MI = microbial inoculation; BC_0_ = zero biochar; BC_1_ = maize-straw synthesized biochar, BC_2_ = cow-manure synthesized biochar; BC_3_ = poultry-manure synthesized biochar; MI_0_ = no microbial inoculation; MI_1_ = Trichoderma harzianum (fungus) microbial inoculation; MI_2_ = Bacillus subtilis (bacteria) microbial inoculation; MI_3_ = combined Trichoderma harzianum and Bacillus subtilis microbial inoculation; Cd_0_ = 0 ppm Cd; Cd_1_ = 10 ppm Cd; Cd_2_ = 30 ppm Cd.

### Soil physicochemical characteristics

**Figure 1:**
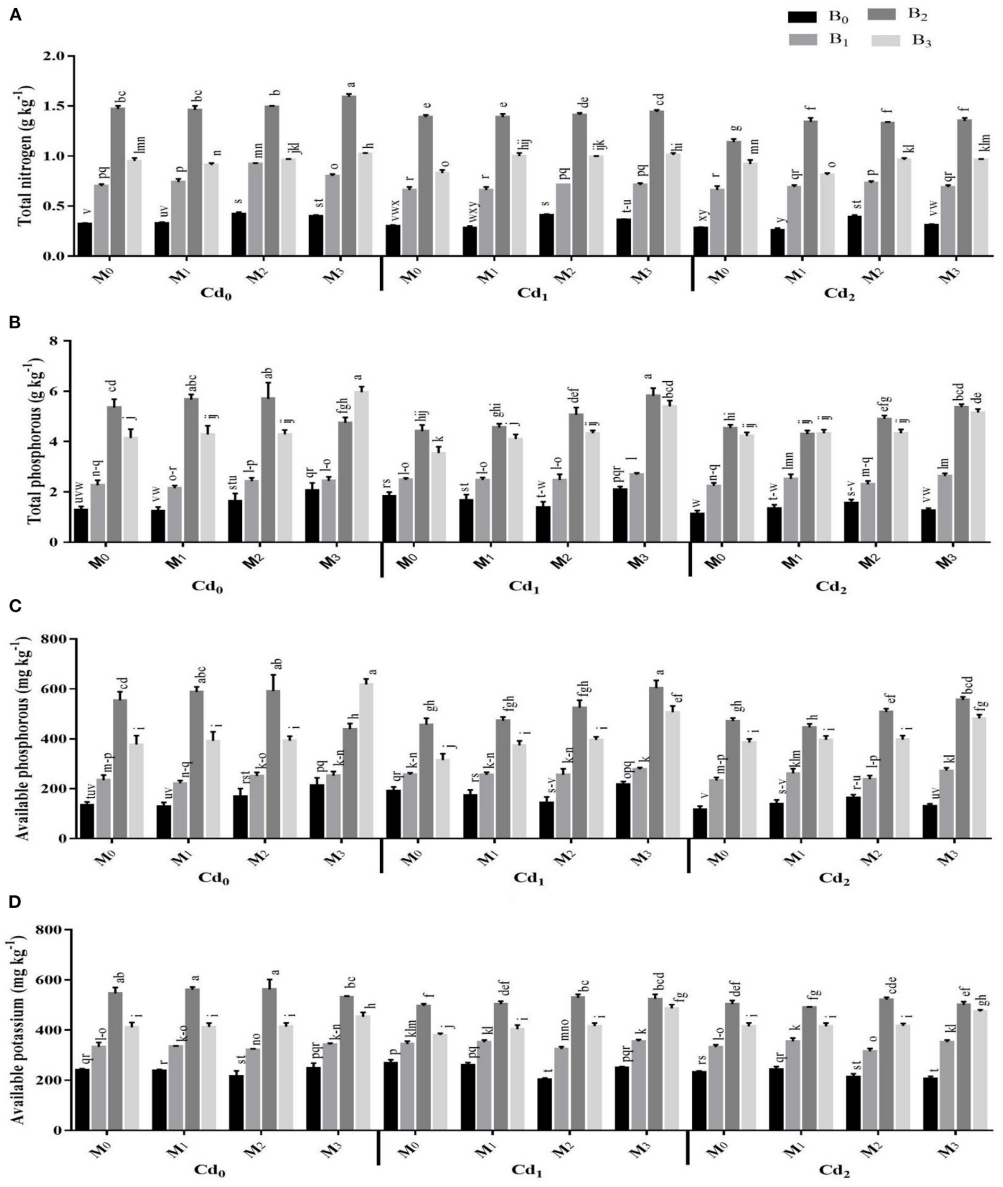
Effect of integrated application of biochar and microbes on **(A)** total nitrogen, **(B)** total phosphorous, **(C)** available phosphorous, and **(D)** available potassium under cadmium (Cd) stress. Bars are representing means ± standard deviation (SD) of three replication. Means within a column followed by the same letters are not significantly different at P ≤ 0.05. BC = biochar types; Cd = artificial cadmium stress; MI = microbial inoculation; BC0 = zero biochar; BC1 = maize-straw synthesized biochar, BC2 = cow-manure synthesized biochar; BC3 = poultry-manure synthesized biochar; MI0 = no microbial inoculation; MI1 = Trichoderma harzianum (fungus) microbial inoculation; MI2 = Bacillus subtilis (bacteria) microbial inoculation; MI3 = combined Trichoderma harzianum and Bacillus subtilis microbial inoculation; Cd0 = 0 ppm Cd; Cd1 = 10 ppm Cd; Cd2 = 30 ppm Cd.

The pre-experiment soil EC and pH were 2.94 mS cm^−1^ and 8.64, respectively. After maize harvest, the interactions between Cd and biochar, Cd and microbes, microbes and biochar and the three-way interaction of Cd, biochar and microbes were significant for the soil physicochemical characteristics (Supplementary Tables S3, S4; Figure 1). With the application of biochar and microorganisms significantly influenced the EC and pH of post-harvested maize soil. The highest organic matter and soil organic carbon were recorded from the application of cow manure (BC_2_) biochar together with combined inoculation of *Trichoderma* and *Bacillus* (MI_3_) without Cd stress. The application of cow manure (BC_2_) biochar had the highest levels of total P, total N, available P, and available K in the soil. However, these were statistically preceded by poultry manure (BC_3_) biochar, and then by maize straw (BC_1_) biochar. Likewise, the microbial inoculation improved the total P, total N, available P, and available K. In this regard, the highest soil total P, total N, available P, and available K were observed in combined inoculation of *Trichoderma* and *Bacillus* (MI_3_).

### Photosynthetic pigments and gas exchange parameters

**Figure 2:**
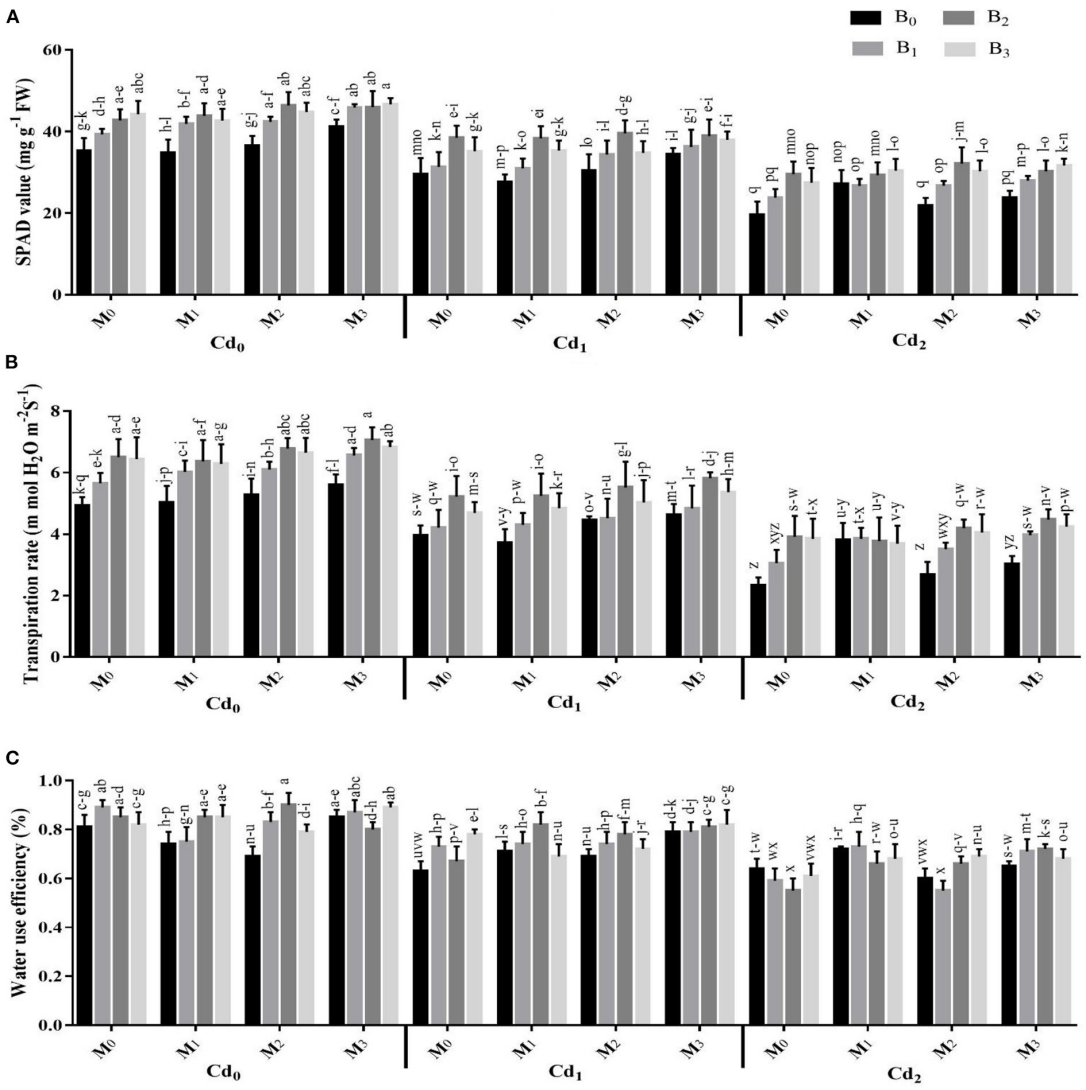
Effect of integrated application of biochar and microbes on **(A)** chlorophyll contents, **(B)** transpiration rate, and **(C)** water use efficiency of maize grown under cadmium (Cd) stress. Bars are representing means ± standard deviation (SD) of three replication. Means within a column followed by the same letters are not significantly different at *P* ≤ 0.05. BC = biochar types; Cd = artificial cadmium stress; MI = microbial inoculation; BC_0_ = zero biochar; BC_1_ = maize-straw synthesized biochar, BC_2_ = cow-manure synthesized biochar; BC_3_ = poultry-manure synthesized biochar; MI_0_ = no microbial inoculation; MI_1_ = Trichoderma harzianum (fungus) microbial inoculation; MI_2_ = Bacillus subtilis (bacteria) microbial inoculation; MI_3_= combined Trichoderma harzianum and Bacillus subtilis microbial inoculation; Cd_0_ = 0 ppm Cd; Cd_1_ = 10 ppm Cd; Cd_2_ = 30 ppm Cd.

**Figure 3:**
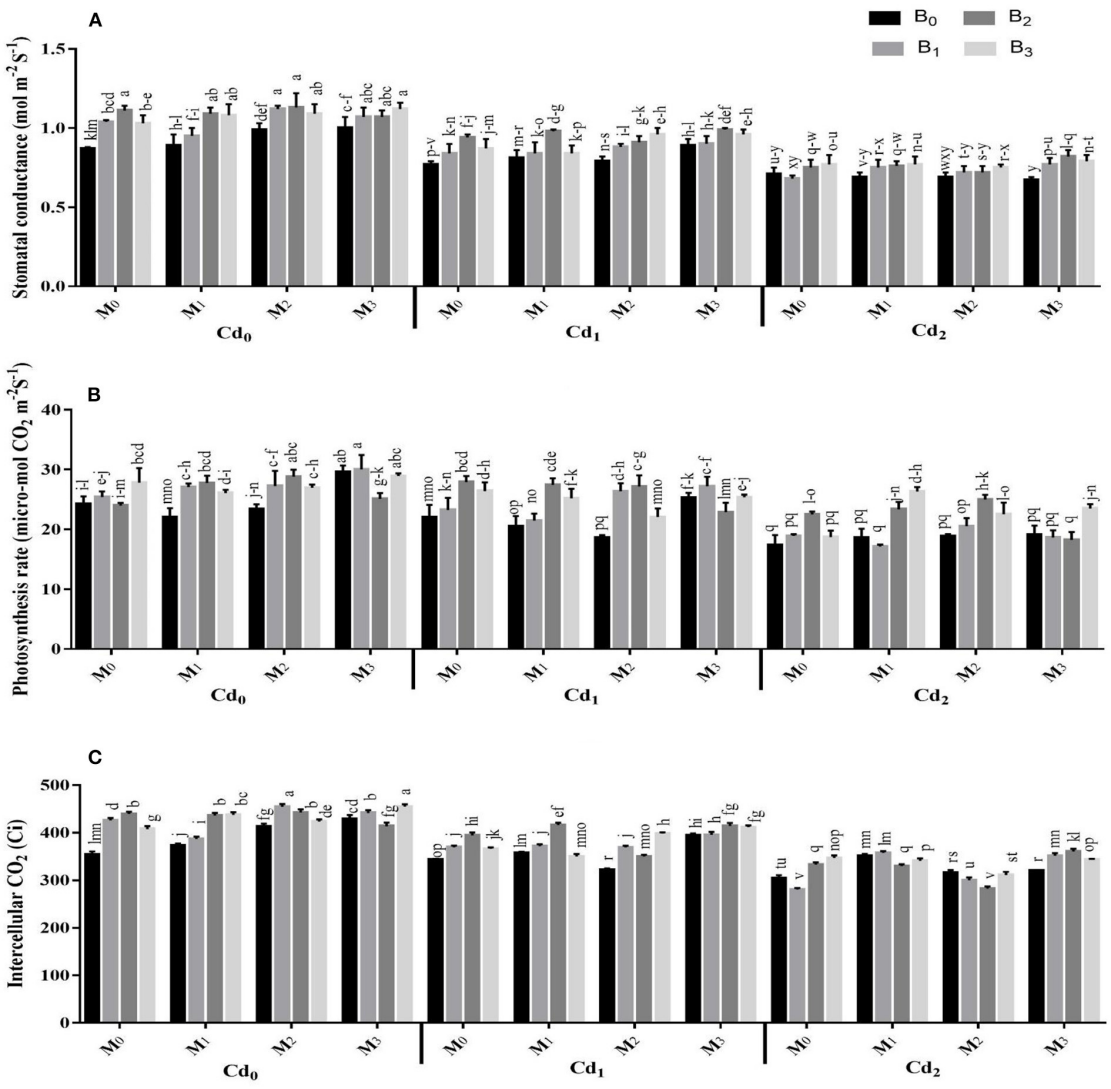
Effect of integrated application of biochar and microbes on **(A)** stomatal conductance, **(B)** photosynthesis rate, and **(C)** intercellular CO_2_ of maize grown under cadmium (Cd) stress. Bars are representing means ± standard deviation (SD) of three replication. Means within a column followed by the same letters are not significantly different at P ≤ 0.05. BC = biochar types; Cd = artificial cadmium stress; MI = microbial inoculation; BC_0_ = zero biochar; BC_1_ = maize-straw synthesized biochar, BC_2_ = cow-manure synthesized biochar; BC_3_ = poultry-manure synthesized biochar; MI_0_ = no microbial inoculation; MI_1_ = Trichoderma harzianum (fungus) microbial inoculation; MI_2_= Bacillus subtilis (bacteria) microbial inoculation; MI_3_ = combined Trichoderma harzianum and Bacillus subtilis microbial inoculation; Cd_0_ = 0 ppm Cd; Cd_1_ = 10 ppm Cd; Cd_2_ = 30 ppm Cd.

Photosynthetic pigments and gas exchange parameters were strongly influenced by the interactions of Cd with biochar, Cd with microbes, microbes with biochar, besides the three-way association between Cd, biochar, and microbes (Supplementary Table S3, Figures 2, 3). The Cd toxicity significantly reduced the chlorophyll SPAD value and gas exchange parameters. The highest reduction in the intercellular CO_2_, SPAD value, transpiration rate, water use efficiency, stomatal conductance, and photosynthesis rate were recorded in Cd_2_ (30 ppm), which were 22.36, 34.50, 40.45, 20.66, 29.07, and 22.41% lower than control, respectively. The addition of biochar in Cd contaminated soil significantly improved the gas exchange parameters and photosynthetic pigments of maize. The highest intercellular CO_2_, SPAD value, transpiration rate, water use efficiency, stomatal conductance, and photosynthesis rate were recorded in cow manure (BC_2_) biochar that was followed by poultry manure (BC_3_) biochar and maize straw (BC_1_) biochar. For microorganism inoculation, the highest intercellular CO_2_, SPAD value, transpiration rate, water use efficiency, stomatal conductance, and photosynthesis rate were recorded in combined microbial inoculants strains (MI_3_) (Figures 2, 3).

### Cd induced oxidative stress and antioxidant activity

**Figure 4:**
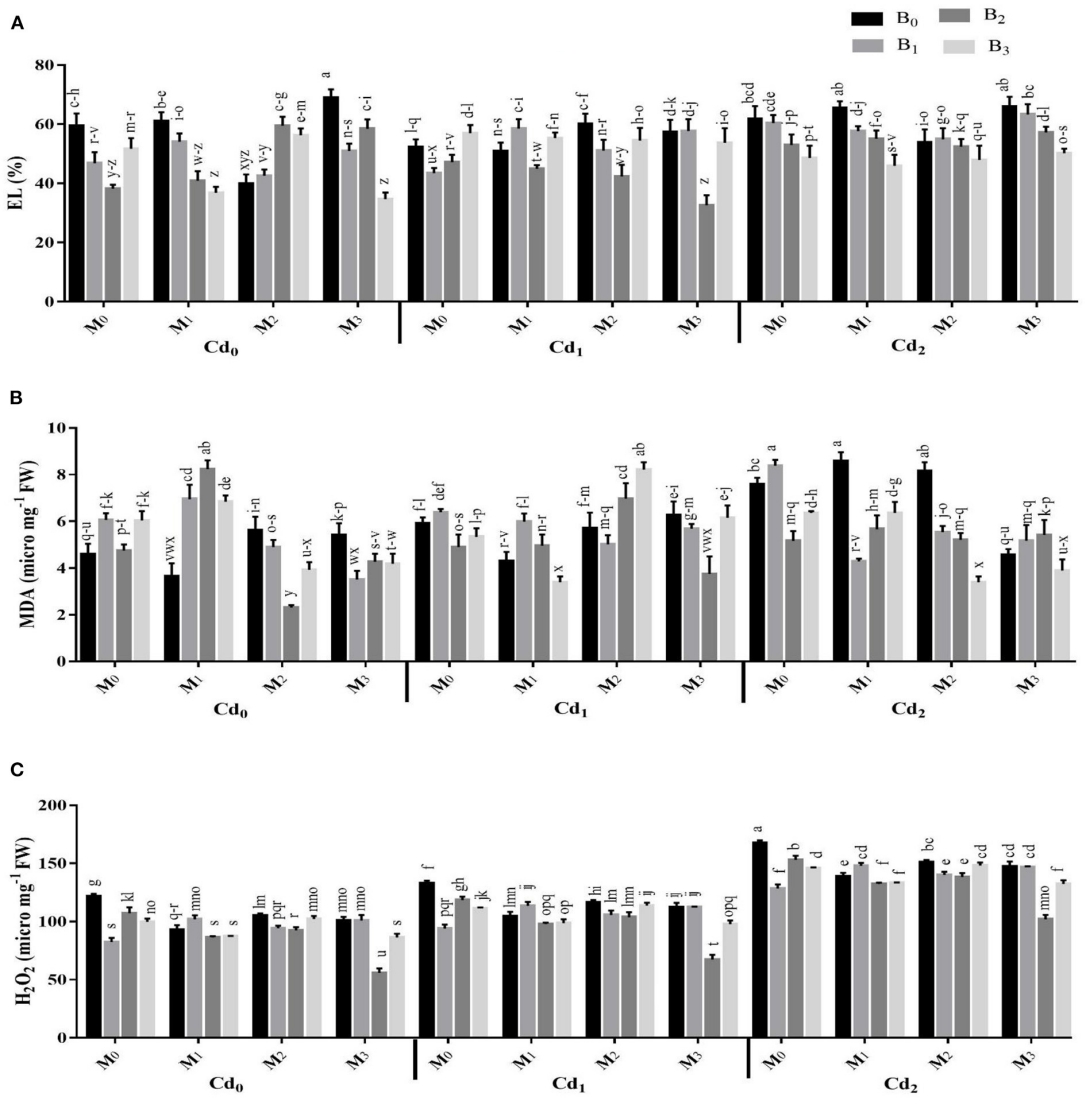
Effect of integrated application of biochar and microbes on **(A)** electrolyte leakage (EL), **(B)** malondialdehyde (MDA), and **(C)** hydrogen peroxide (H_2_O_2_) of maize grown under cadmium (Cd), stress. Bars are representing means ± standard deviation (SD) of three replication. Means within a column followed by the same letters are not significantly different at P ≤ 0.05. BC = biochar types; Cd = artificial cadmium stress; MI = microbial inoculation; BC_0_ = zero biochar; BC_1_ = maize-straw synthesized biochar, BC_2_ = cow-manure synthesized biochar; BC_3_ = poultry-manure synthesized biochar; MI_0_ = no microbial inoculation; MI_1_ = Trichoderma harzianum (fungus) microbial inoculation; MI_2_= Bacillus subtilis (bacteria) microbial inoculation; MI_3_ = combined Trichoderma harzianum and Bacillus subtilis microbial inoculation; Cd_0_ = 0 ppm Cd; Cd_1_ = 10 ppm Cd; Cd_2_ = 30 ppm Cd.

**Figure 5:**
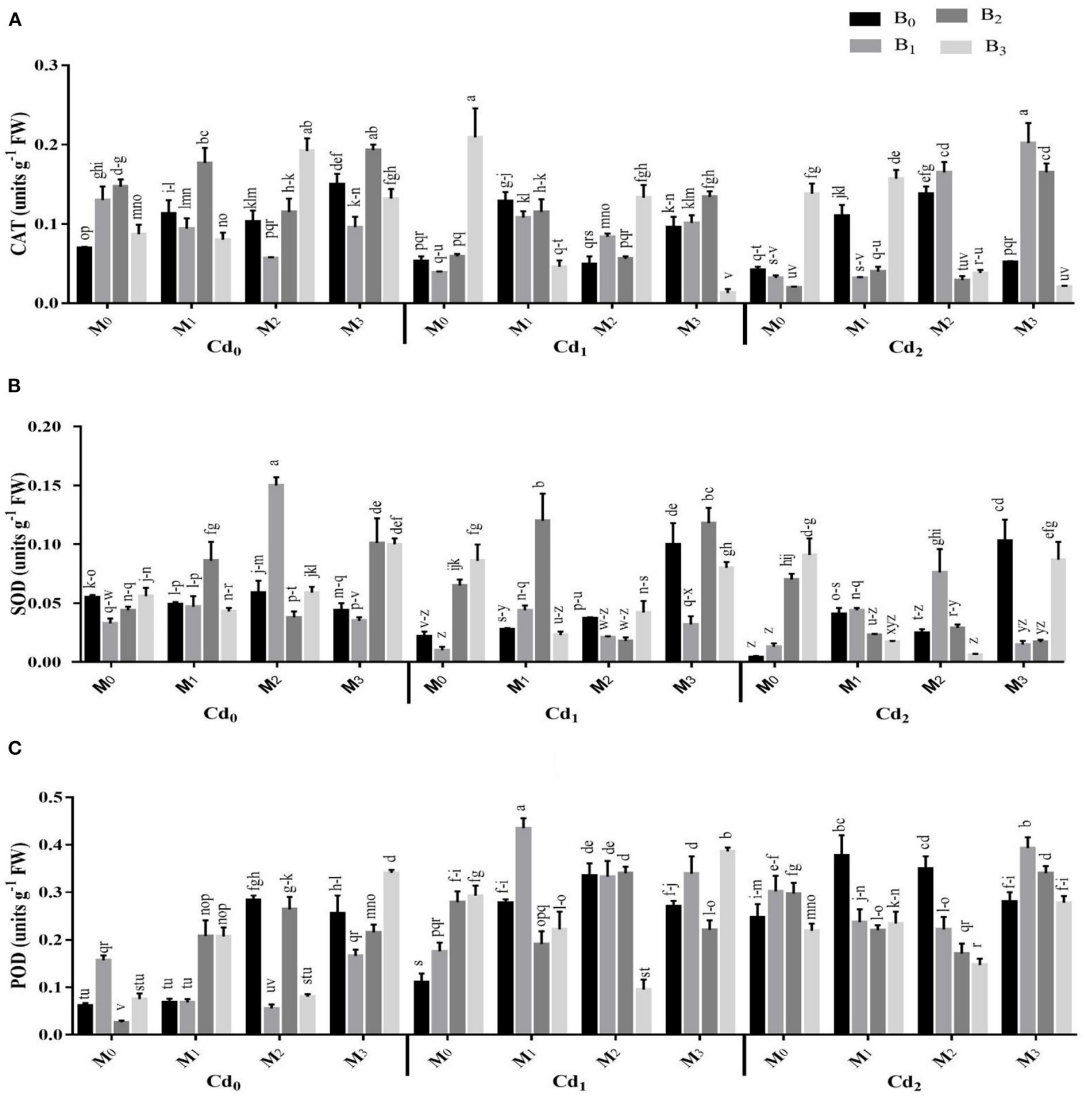
Effect of integrated application of biochar and microbes on activities of **(A)** catalase (CAT), **(B)** superoxide dismutase (SOD), and **(C)** peroxidase (POD), of maize under cadmium (Cd) stress. Bars are representing means ± standard deviation (SD) of three replication. Means within a column followed by the same letters are not significantly different at P ≤ 0.05. BC = biochar types; Cd = artificial cadmium stress; MI = microbial inoculation; BC_0_ = zero biochar; BC_1_ = maize-straw synthesized biochar, BC_2_= cow-manure synthesized biochar; BC_3_ = poultry-manure synthesized biochar; MI_0_ = no microbial inoculation; MI_1_ = Trichoderma harzianum (fungus) microbial inoculation; MI_2_ = Bacillus subtilis (bacteria) microbial inoculation; MI_3_ = combined Trichoderma harzianum and Bacillus subtilis microbial inoculation; Cd_0_ = 0 ppm Cd; Cd_1_ = 10 ppm Cd; Cd_2_ = 30 ppm Cd.

The Cd stress and application of biochar microbial inoculation and three-way interaction significantly affected the oxidative damages and activities of antioxidant enzymes (Figures 4, 5). Results highlighted that Cd stress caused significant increase in electrolyte leakage (EL), malondialdehyde (MDA), and hydrogen peroxide (H_2_O_2_) (Figure 4). The highest EL of the maize leaves was observed with Cd-spiked soil inoculated with combined inoculants strains (MI_3_), having an average value of 68.84 ± 2.88%, which was 13.67% higher than control. The highest MDA contents were observed maximum in artificial Cd-spiked soil (30 ppm) inoculated with *Trichoderma harzianum* strain (MI_1_), having an average value of 8.57 ± 0.39 μM g^−1^ FW, that was 46.56% greater than control. Correspondingly, the highest concentration of H_2_O_2_ was observed in high Cd spiked soil (Cd_2_BC_0_MI_0_), having a mean value of 167.44 ± 2.29, which was 27.47% higher than control. However, the biochar application caused significant reduction in oxidative damages. The highest reduction in EL, MDA, and H_2_O_2_ were recorded from cow manure (BC_2_) biochar, which was 16.50, 12.37, and 15.83% respectively lower than the control The combined inoculation of microbes in Cd contaminated soil also reduced the EL, MDA, and H_2_O_2_ in maize leaves. The Cd stress caused significant decrease in the activities of CAT and SOD whereas the POD activity was significantly improved compared with control. The application of biochar application significantly enhanced the actities of CAT and SOD and caused significant reduction in the POD activity under Cd stress. The highest activities of CAT and SOD were noted from cow manure (BC_2_) biochar. Combined inoculation of microorganisms (MI_3_) also caused significant improvement in the activities of CAT, SOD, and POD.

### Concentration of Cd in soil and plant parts

The three-way interaction of Cd, microbes, and biochar significantly influenced by Cd concentration in soil and various maize parts i.e., shoot and root (Table 3). The soil Cd concentration was higher than that of shoot and root at all levels of Cd contamination (Table 3). The application of biochar caused significant decrease in shoot and root Cd. In this regard, the highest reduction was noted from cow manure (BC_2_) biochar that was followed by poultry manure (BC_3_) biochar and maize-straw (BC_1_) biochar. The inoculation of soil microorganisms also caused significant reduction in in shoot and root Cd.

**Table 3:** Effect of integrated application of biochar and microbes on cadmium (Cd)-concentration in soil, shoot, and root of maize under Cd stress.

**Cd**	**MI**	**BC_0_**	**BC_1_**	**BC_2_**	**BC_3_**	**Mean (Cd × MI)**
Soil cadmium (mg kg^−1^ DW)						
Cd_0_	MI_0_	0.50 ± 0.02 r	0.41 ± 0.03 r	0.53 ± 0.02 r	0.47 ± 0.04 r	0.48 ± 0.05 H
	MI_1_	0.47 ± 0.03 r	0.48 ± 0.05 r	0.55 ± 0.04 r	0.80 ± 0.04 r	0.58 ± 0.16 GH
	MI_2_	0.69 ± 0.03 r	0.92 ± 0.05 r	0.76 ± 0.02 r	0.73 ± 0.02 r	0.77 ± 0.10 G
	MI_3_	0.76 ± 0.06 r	0.70 ± 0.03 r	0.78 ± 0.02 r	0.79 ± 0.05 r	0.76 ± 0.04 G
Cd_1_	MI_0_	7.46 ± 0.11 no	6.76 ± 0.12 p	7.10 ± 0.24 op	8.54 ± 0.29 m	7.46 ± 0.77 F
	MI_1_	6.18 ± 0.39 q	8.79 ± 0.24 lm	6.80 ± 0.23 p	8.73 ± 0.39 lm	7.62 ± 1.33 F
	MI_2_	8.63 ± 0.27 m	6.91 ± 0.16 p	9.24 ± 0.49 kl	9.71 ± 0.13 k	8.62 ± 1.23 D
	MI_3_	7.66 ± 0.30 n	9.71 ± 0.28 k	7.50 ± 0.16 no	7.67 ± 0.23 n	8.13 ± 1.05 E
Cd_2_	MI_0_	17.76 ± 0.78 e	16.22 ± 0.30 fg	20.59 ± 0.45 b	18.70 ± 0.52 d	18.32 ± 1.83 A
	MI_1_	14.98 ± 0.34 h	14.76 ± 0.20 h	19.38 ± 0.55 c	17.38 ± 0.52 e	16.62 ± 2.19 B
	MI_2_	13.67 ± 0.53 i	14.79 ± 0.64 h	22.31 ± 0.60 a	14.80 ± 0.56 h	16.39 ± 3.98 B
	MI_3_	11.96 ± 0.29 j	16.65 ± 0.25 f	14.19 ± 0.49 i	15.81 ± 0.59 g	14.65 ± 2.06 C
Mean (BC)		7.56 ± 6.12 D	8.09 ± 6.45 C	9.14 ± 8.18 A	8.68 ± 6.88 B	
LSD (p 0.05) value	Cd × MI = 0.26; BC = 0.16; Cd × MI × BC = 0.54					
Root cadmium (mg kg^−1^ DW)						
Cd_0_	MI_0_	1.07 ± 0.26 n	0.79 ± 0.05 n	0.92 ± 0.16 n	0.76 ± 0.07 n	0.89 ± 0.14 F
	MI_1_	1.10 ± 0.42 n	0.85 ± 0.21 n	0.79 ± 0.25 n	0.58 ± 0.07 n	0.83 ± 0.22 F
	MI_2_	0.97 ± 0.09 n	0.75 ± 0.12 n	0.44 ± 0.07 n	0.58 ± 0.12 n	0.68 ± 0.23 F
	MI_3_	1.27 ± 0.36 n	0.80 ± 0.15 n	0.56 ± 0.14 n	0.44 ± 0.10 n	0.77 ± 0.37 F
Cd_1_	MI_0_	7.05 ± 0.59 g	4.53 ± 0.40 kl	5.08 ± 0.42 jkl	5.47 ± 0.46 jk	5.53 ± 1.08 D
	MI_1_	4.44 ± 0.41 kl	5.26 ± 0.47 jk	3.32 ± 0.60 m	5.61 ± 0.33 ij	4.66 ± 1.02 E
	MI_2_	6.95 ± 0.75 g	6.74 ± 0.73 gh	7.05 ± 0.59 g	5.86 ± 0.42 hij	6.65 ± 0.54 C
	MI_3_	10.72 ± 0.06 cd	7.13 ± 1.47 g	1.49 ± 0.28 n	4.16 ± 0.33 lm	5.87 ± 3.97 D
Cd_2_	MI_0_	15.40 ± 0.25 a	7.47 ± 1.27 g	10.10 ± 0.52 cde	9.68 ± 1.38 def	10.66 ± 3.36 AB
	MI_1_	12.90 ± 0.66 b	9.38 ± 1.31 ef	8.71 ± 0.29 f	10.30 ± 1.45 cde	10.32 ± 1.84 B
	MI_2_	12.39 ± 0.78 b	9.80 ± 0.47 de	10.98 ± 1.14 c	10.70 ± 0.89 cd	10.97 ± 1.07 A
	MI_3_	10.68 ± 0.93 cd	6.61 ± 0.45 ghi	6.57 ± 1.02 ghi	4.13 ± 1.04 lm	7.00 ± 2.71 C
Mean (BC)		7.08 ± 5.29 A	5.01 ± 3.43 B	4.67 ± 3.94 C	4.86 ± 3.85 BC	
LSD (p 0.05) value	Cd × MI = 0.53; BC = 0.31; Cd × MI × BC = 1.06					
Plant shoot cadmium (mg kg^−1^ DW)						
Cd_0_	MI_0_	0.85 ± 0.07 rst	0.34 ± 0.02 uvw	0.30 ± 0.18 uvw	0.27 ± 0.07 uvw	0.44 ± 0.27 H
	MI_1_	0.46 ± 0.10 t-w	0.20 ± 0.13 vw	0.47 ± 0.10 t-w	0.58 ± 0.08 s-v	0.43 ± 0.16 H
	MI_2_	0.64 ± 0.03 s-v	0.02 ± 0.01 w	0.19 ± 0.09 vw	0.57 ± 0.04 s-v	0.36 ± 0.30 H
	MI_3_	1.24 ± 0.02 qr	0.74 ± 0.15 stu	0.43 ± 0.06 t-w	0.36 ± 0.04 uvw	0.69 ± 0.40 G
Cd_1_	MI_0_	2.44 ± 0.20 klm	2.07 ± 0.10 mno	2.28 ± 0.46 lm	2.75 ± 0.34 k	2.39 ± 0.29 E
	MI_1_	3.36 ± 0.07 j	2.77 ± 0.07 k	2.35 ± 0.16 klm	1.66 ± 0.24 opq	2.53 ± 0.72 E
	MI_2_	3.42 ± 0.36 j	1.69 ± 0.27 opq	2.19 ± 0.34 mn	2.21 ± 0.21 mn	2.38 ± 0.74 E
	MI_3_	1.55 ± 0.15 pq	1.77 ± 0.25 nop	2.68 ± 0.31 kl	0.99 ± 0.15 rs	1.75 ± 0.70 F
Cd_2_	MI_0_	8.98 ± 0.46 c	11.62 ± 0.57 a	5.78 ± 0.30 f	8.17 ± 0.27 d	8.64 ± 2.41 A
	MI_1_	4.15 ± 0.20 hi	7.36 ± 0.41 e	3.77 ± 0.23 ij	7.67 ± 0.49 e	5.74 ± 2.06 C
	MI_2_	5.28 ± 0.56 g	5.14 ± 0.30 g	5.06 ± 0.54 g	4.38 ± 0.13 h	4.97 ± 0.40 D
	MI_3_	10.78 ± 0.37 b	5.75 ± 0.36 f	3.60 ± 0.43 j	5.07 ± 0.59 g	6.30 ± 3.12 B
Mean (BC)		3.60 ± 3.32 A	3.29 ± 3.54 B	2.43 ± 1.88 D	2.89 ± 2.82 C	
LSD (p 0.05) value	Cd × MI = 0.23; BC = 0.13; Cd × MI × BC = 0.46					

Values are representing means ± standard deviation (SD) of three replication. Means within a column sharing the same letters do not differ significantly different at P ≤ 0.05. BC = biochar types; Cd = artificial cadmium stress; MI = microbial inoculation; BC_0_ = zero biochar; BC_1_ = maize-straw synthesized biochar, BC_2_ = cow-manure synthesized biochar; BC_3_ = poultry-manure synthesized biochar; MI_0_ = no microbial inoculation; MI_1_ = Trichoderma harzianum (fungus) microbial inoculation; MI_2_ = Bacillus subtilis (bacteria) microbial inoculation; MI_3_ = combined Trichoderma harzianum and Bacillus subtilis microbial inoculation; Cd_0_ = 0 ppm Cd; Cd_1_ = 10 ppm Cd; Cd_2_ = 30 ppm Cd.

### Correlation matrix

**Figure 6:**
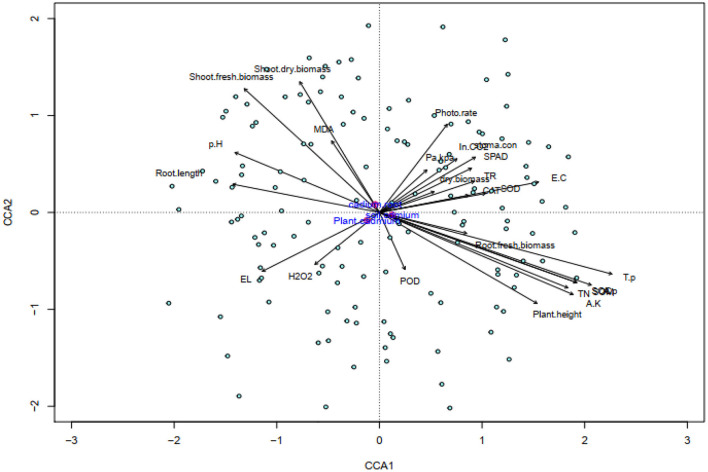
Canonical correspondence analysis (CCA), of soil physico-chemical properties and maize plant physiological traits, showing the contribution of Cd toxicity, biochar and microbes addition to variability. Phote. rate, photosynthesis rate; Pa/kPr, water use efficiency; SPAD, chlorophyll SPAD value; TR, transpiration rate; SC, stomatal conductance; total nitrogen, T.N; total phosphorous, T.P; available phosphorous, A.P; available potassium, A.P; In CO_2_, Intercellular carbon dioxide; RFB, root fresh biomass; RDB, root dry biomass; RL, root length; PH, plant shoot height; SFB, plant shoot fresh biomass; SDB, plant shoot dry biomass; catalase, CAT; superoxide dismutase, SOD; peroxidase, POD; electrolyte leakage, EL; malondialdehyde, MDA; hydrogen peroxide, H_2_O_2_; P_Cd, cadmium concentration in plant shoot; cadmium; S_Cd, cadmium concentration in soil; R_Cd, cadmium concentration in plant root.

Three clusters showed the response variables that indicated a strong positive correlation with each other. The left top cluster showed the correlation strength of Cd concentration in root, soil, and shoots part. An increase in soil and plant Cd was negatively correlated with plant growth and gas exchange parameters. The CCA was done to assess the extent of different parameters of plant's physiological processes affected by Cd uptake of different plant parts of maize. The first component (horizontal axis) explained 35.82% variation (eigenvalue 0.01014, *p* = 0.001^***^), while second component was 15.62% (eigenvalue 0.004421, *p* = 0.001^***^). Their cumulative results show the 51.4% inertia which is significant at a 0.001 significance level, indicating good correlations between cadmium uptake, growth, and physiological parameters of maize plant (Figure 6).

### Microscopic characterization of biochar

**Figure 7:**
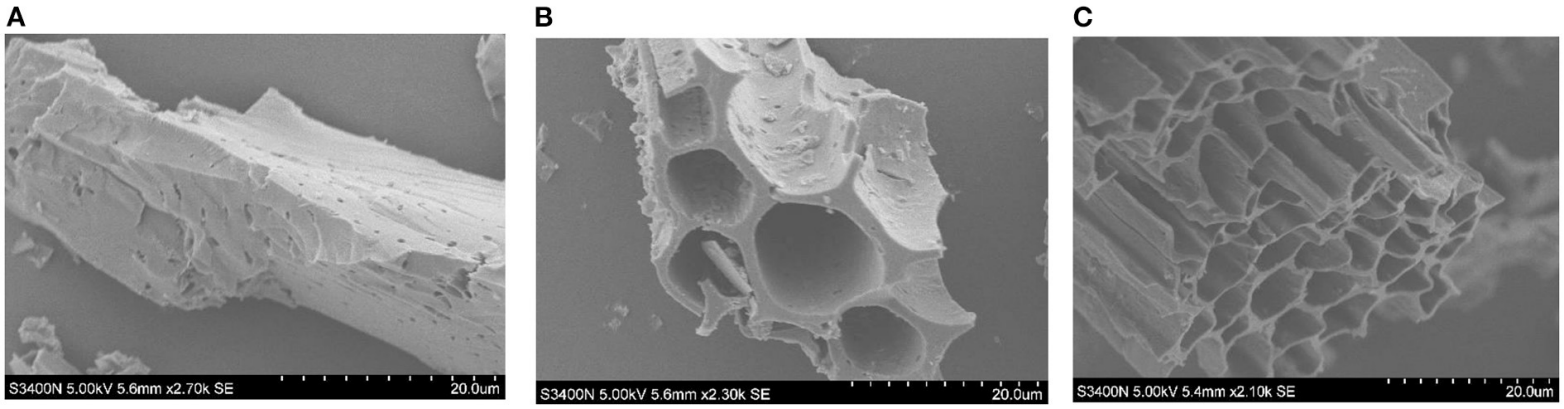
SEM (Scanning electron microscopy) images of BC_1_ maize-straw biochar **(A)**, BC_2_ cow-manure biochar **(B)**, and BC_3_ poultry-manure biochar **(C)** which were produced at 550°C temperature (scale for measuring SEM was 20.0 μm).

The microscopic images revealed that there were noticeable variations in different biochar samples (Figure 7). Biochar particles were randomly distributed, as seen in the photographs, demonstrating the heterogeneous composition of the pyrolyzed materials. Biochar derived from maize straw (BC_1_) was more abundant in amounts of aromatic structures and higher amorphous mass disorder, whereas biochar derived from animal (BC_2_) and poultry (BC_3_) manures were having rising sheets of conjugated aromatic carbon structures pleated over each other in a turbo-statically arrangement (Figure 7).

## Discussion

The findings of this study confirmed the hypothesis that combined application of microbes [*Trichoderma harzianum* (fungus) and *Bacillus subtilis* (bacteria)] inoculation, and biochar significantly influenced the physicochemical characteristics of soil, improved maize performance, and reduced Cd bioavailability and stabilization in Cd contaminated soil. The Cd toxicity caused significant reduction in root and shoot growth, and overall biomass of maize (Tables 1, 2; Figures 2, 3).

Cadmium causes chlorophyll destruction, restricts nutrient uptake, and reduces photosynthetic activity resulting in significant reduction in plant growth (Abbas et al., 2018; Zhang et al., 2019; Farooq et al., 2020). Reduction in the uptake of the essential nutrients, physiological and morphological traits of the plant is regulated by Cd which can be transferred to the aboveground parts of the plant by replacing essential ions i.e., Ca due to having the same chemical behavior, ionic radius, and charge (Haider et al., 2021; Zulfiqar et al., 2022). This may directly hamper metabolic processes, resulting in growth inhibition, and yield reduction (Abbas et al., 2017; El-Naggar et al., 2020). The biochar having high adsorption potential helped to ameliorate the toxicity of Cd (Younis et al., 2016), and improved root morphological characters i.e., root length and biomass, which enhanced the shoot biomass of maize eventually (Tables 1, 2). The soil used in the current study was calcareous (Supplementary Table S1), therefore, biochar-induced soil alkalinity restricted nutrient availability (Zulfiqar et al., 2019). The soil of current study was a complete mixture of loam and sand having alkaline pH of 8.64.

The present study showed that soil nutrients were increased by biochar incorporation in Cd-contaminated soil (Supplementary Table S4; Figure 1). This may be due to the appropriate application of synthetic fertilizers and biochar, which helps in improving the soil organic matter and nutrients availability in the Cd contaminated rhizosphere soil. Furthermore, soil physical properties i.e., porosity, aggregation capacity, and water storage capacity were greatly enhanced by the addition of biochar in Cd-contaminated soil (Yuan et al., 2018). Improvement of soil properties may improve soil fertility by increasing the availability of nutrients, and reducing the nutrients leaching (Abbas et al., 2018; Haider et al., 2022b). Similarly, the addition of biochar in Cd-polluted agricultural soils results into improvement of essential nutrient contents P, C, K, N, and Ca in soil which further improved the plant biomass (Abbas et al., 2017; Bashir et al., 2017). Biochar is a renewable porous carbonaceous resource loaded with phosphate, nitrate, and ammonium that enhances the fertility status of agricultural soil (Rehman et al., 2016). Biochar can also be utilized as a source of nutrients to improve the fertility status of soil in various agronomic crops i.e., rice (*Oryza sativa* L.), wheat (*Triticum aestivum* L.), maize (*Zea mays* L.), alfalfa (*Medicago sativa* L.), and soybean (*Glycine max* L.), due to the inclusion of soluble nutrients and the labile fraction of biochar comprising organically bound nutrients for mineralization (Sabae et al., 2006; Abbas et al., 2017; Zulfiqar et al., 2022).

Photosynthetic activity is regarded as an important index for determining the damages at cellular level caused by Cd toxicity in plants (Gallego et al., 2012; Farhangi-Abriz and Torabian, 2016). The current study showed that contamination of Cd results in a decrease in maize growth, photosynthesis pigments, and gas exchange observations and the reduction of these observations escalated with a higher Cd-concentration in contaminated soil (Figures 2, 3). The reduction in photosynthesis activity, pigments, and gas exchange parameters in many horticultural and agronomics crops (Mohsenzadeh and Shahrokhi, 2014; Younis et al., 2016), maybe due to ultra-structural alternations in Cd-induced plants and a decrease in plant nutrients uptake (Zhang et al., 2019). It was reported that oxidative stress affects various plant metabolic processes and cellular functions and causes nucleic acids disruption (Haider et al., 2021; Zulfiqar et al., 2022). In the current study, toxic Cd concentration enhanced the activities of EL, H_2_O_2_, POD, and MDA while decreasing the activities of SOD and CAT in maize leaves as compared plants treated with no Cd contamination (Figures 4, 5). The increase in oxidative stress and the decrease in the SOD and CAT synthesis under higher toxic Cd concentration in leaves may be attributed to the reduced anti-oxidative capacity of maize due to Cd toxicity. Furthermore, results indicate that the application of biochar as an amendment in Cd-contaminated soil significantly decreased the activities of EL, MDA, H_2_O_2_, and POD, while improving the SOD and CAT synthesis activities of maize leaves as compared with control (Figures 4, 5). In another study, Farooq et al. (Farooq et al., 2020) reported that higher contamination of Cd in agricultural soils significantly regulates the antioxidant and oxidant activities of various crops i.e., castor (*Ricinus communis* L.), mustard (*Brassica juncea* L.), wheat, maize, and rice (Farhangi-Abriz and Torabian, 2016; Haider et al., 2021). Application of biochar in Cd-contaminated soil reduced the bioavailability of Cd to plants which reduces the synthesis of H_2_O_2_ and EL as compared to soil contaminated with Cd and having no biochar (Abbas et al., 2018). Similarly in another study, incorporation of biochar significantly minimized the activity of MDA in spinach (*Spinacia oleracea* L.) leaves cultivated in Cd-contaminated soil (Younis et al., 2016).

Recent literature highlighted that application of biochar in contaminated soil/water had significant potential to absorb anthropogenic toxins i.e., trace metals, petroleum hydrocarbons, steroid hormones, and other organic contaminants, from water or soil (Ding et al., 2016). Biochar has possessed aromatic hydrocarbon structure, high pH, active surface functional groups and porous structure (El-Naggar et al., 2020). These features of biochar play a crucial role in the remediation mechanism of organic and inorganic pollutants by precipitation, complexation, electrostatic interaction, ion exchange, and physical adsorption (Shaaban et al., 2018). Furthermore, the adsorption of biochar is largely dependent on its large specific surface area, microporous structure, active functional group, and pH which can further be modified by a variation in pyrolysis temperature, retention time, and feedstock used for biochar preparation (Aebi, 1984). The SEM graphics of biochar pyrolyzed from feedstock i.e., maize straw showed the observable plant structure residues, for example degraded cellulose and lignin due to high pyrolysis temperature such as 550°C. The numerous pyrolysis processes of the parent feedstock are produced by the variation and heterogeneity in the composition of biochars. In our study, the SEM graphics of different biochar materials clearly show the distinct structural composition of biochar. The cellulose structure of maize straw biochar can be classified as prismatic, fibrous, or spherical because there are visible and possibly associated signs of porous and fibrous longitudinal structures of plant cell walls (Rasul et al., 2016). In addition, the biochar processed from cow and poultry manure depicts differences in the coarseness (macropores in cow manure biochar and micropores in poultry manure biochar), whereas small pores and sand micro-particles having uneven porosity substantially improved soil health and plant yield. The deviation and difference in the feedstock's absorbent composition may have a substantial effect on the adsorption potential of trace metals remediation in metal contaminated agricultural soils by biochar (Bashir et al., 2017; Abbas et al., 2018).

Remediation of Cd contaminated soils can be accomplished using, biological, physical, and chemical approaches. Physical and chemical remediation methods provide quick remediation but are costly and cause secondary pollutants (Zhang et al., 2019). Microbial remediation depends on rhizosphere competent microbial flora and root exudates secreted by plants and already localized rhizosphere microbes. Plant roots secrete exudates including organic acids, alcohols, and sugars that serve as a source of energy in the form of carbohydrates for the microflora of soil and improve microbial activity and growth (Yaghoubian et al., 2019). Certain root exudates may also serve as chemotactic signals for microbial flora. Additionally, plant roots help to loosen the soil structure and improve water transport in the rhizosphere, thereby further boosting microbial activities (Chellaiah, 2018). Microorganism survival in the presence of toxic metals in soil depends on structural and biochemical properties, genetic and/or physiological adaptation, modification of trace metals in the environment, and its specification, toxicity, and availability (Ahmad et al., 2013; Hammer et al., 2015). Microorganism's inoculation in trace metal contaminated agricultural soil greatly remediates the toxicity of Cd and enhanced the plants growth and photosynthesis activity (Lata et al., 2019).

Inoculation of *Trichoderma harzianum* (fungi) and *Bacillus subtilis* (bacteria) in Cd-contaminated maize soil, has been observed to significantly minimize the toxicity of Cd in the root zone of maize and enhance the root growth of crops like mustard, soybean, alfalfa, wheat, and rice (Han et al., 2016). Previous research has shown that inoculation of *Bacillus* sp., can significantly amend the contamination of trace metals from urban waste and industrial effluents by accompanying mechanisms i.e., biosorption, biotransformation, biomineralization, and bioaccumulation (Ejaz et al., 2021). Similarly, inoculation of *Pseudomonas* sp, was also effective for appropriate bioremediation of Cu, Cd, Cr, Hg, Ni, Pb, U, and Zn (Mehmood et al., 2017). To mitigate the toxic effect of Cd in the soil, microbes have adopted a series of pathways such as (1) by metal ions pumping exterior the cell membrane (Ahmad et al., 2014), (2) inside accumulation and cell sequestration of trace metal ions (Lata et al., 2019), (3) conversion/transformation of toxic trace metals into a less toxic form (Yaghoubian et al., 2019), and (4) adsorption/desorption of toxic metals (Zhang et al., 2019). Current experiment observations revealed that integration of microorganism's inoculation and biochar was considered more valuable in improving the maize growth under Cd contaminated soil as contrasted to sole application of biochar or microbes. Biochar is porous in nature which serves as a habitat for the optimum survival of microorganisms in the rhizosphere (Zhang et al., 2018). The coupling of both microorganisms and biochar in contaminated soil may provide a habitat for microorganisms in the soil that significantly affects the metabolic processes in the plant directly or indirectly (Haider et al., 2022c), and further leads to enhance the microbe population with respect to microbial density and abundance (Zulfiqar et al., 2022). In addition, biochar can increase the soil's cation exchange ability and conserve nutrients for microbial growth by the absorption of nutrient cations from biochar functional groups (Hussain et al., 2021). Moreover, the porous structure of biochar helps in the adsorption of trace metal ions in it those are further modified into non-toxic form by the activity of metal remediating-microorganisms in soil (Yaghoubian et al., 2019). Integrated addition of biochar with microorganisms i.e., *Trichoderma harzianum* (fungi) and *Bacillus subtilis* (bacteria) enhanced the remediation potential in soil against contaminants like Cd because of positive synergistic response of biochar on microorganisms' growth (Zhang et al., 2019; Zhou et al., 2019). In the present study, the improvement with the intermixed treatment of biochar and microorganisms might be due to the same mechanisms. An adequate application of biochar can improve the soil properties i.e., water retention, cation exchange capacity, aeration conditions, and pH which enhance the growth of microorganisms in the rhizosphere contaminated with trace metals, which is a major component of bioremediation in trace metal contaminated agricultural soils (Yuan et al., 2018). In contrast to this experiment, Cui et al. (2016) found that the efficiency of the biochar treatment decreased with time due to biochar aging and alkalinity. A decrease in soil pH and an increase in trace-metal bioavailability were detected because of alkalinity leaching (Haider et al., 2022c; Qianqian et al., 2022). To better understand biochar aging in the field, as well as the influence of variables like alkalinity leaching, more research on the simultaneous application of biochar and microbes in diverse climatic zones and soil settings is needed.

## Conclusion

Combined application of cow manure biochar and microorganism's inoculations in maize grown in Cd-contaminated soil significantly influenced the bioavailability of Cd, improved soil physicochemical properties, antioxidant activity, photosynthesis, and growth morphology of maize. Further studies are required to evaluate the potential of both biochar and microorganisms in improving plant growth, and its effects on the accumulation of trace metals in plants that are simultaneously affected by many trace metals under trace metals-contaminated agricultural soils. Future studies should be based on long-term field experiments in different soil environments and climatic zones.

## Data availability statement

The original contributions presented in the study are included in the article/Supplementary material, further inquiries can be directed to the corresponding author.

## Author contributions

Conceptualization: FH and CL. Methodology and writing—original draft preparation: FH. Software: NA. Validation: MF and SC. Formal analysis: MN and MS. Writing—review and editing: MF, SC, and AM. Supervision and funding acquisition: CL. All authors have read and agreed to the published version of the manuscript.

## Funding

This research was financially supported by the National Key R&D Program of China (2021YFD1900700).

## Conflict of interest

The authors declare that the research was conducted in the absence of any commercial or financial relationships that could be construed as a potential conflict of interest.

## Publisher's note

All claims expressed in this article are solely those of the authors and do not necessarily represent those of their affiliated organizations, or those of the publisher, the editors and the reviewers. Any product that may be evaluated in this article, or claim that may be made by its manufacturer, is not guaranteed or endorsed by the publisher.
