# Supplementary material for: Influence of biochar and microorganism co-application on stabilization of cadmium (Cd) and improved maize growth in Cd-contaminated soil
Source: Front Plant Sci. 2022 Sep 8;13:983830. doi: 10.3389/fpls.2022.983830 (PMC9493347; doi:10.3389/fpls.2022.983830)
Supplement: Supplementary file 1 [file Data_Sheet_1.docx]

**Table S1**: Physiochemical properties of different biochars used in the experiment

| Parameters | Maize straw | Cow manure | Poultry manure |
| --- | --- | --- | --- |
| pH | 8.6 | 7.07 | 7.53 |
| EC (mS cm^-1^) | 3.78 | 5.86 | 4.72 |
| Total N (g kg^-1^) | 0.1441 | 0.149 | 0.147 |
| Total K % | 17.3 | 34.8 | 32.9 |
| Total P % | 0.189 | 0.697 | 0.429 |
| Total C (g kg^-1^) | 6.7 | 7.9 | 7.3 |

EC = electrical conductivity; N = nitrogen; K= potassium; P = phosphorous; C = carbon

**Table S2**: Experimental treatments

| Factor 1 (Biochar) | Factor 2 (Microorganisms) | Factor 3 (Cadmium) |
| --- | --- | --- |
| No biochar (BC_0_) | No inoculant (MI_0_) | 0 ppm (Cd_0_) |
| Maize biochar (BC_1_) | *Trichoderma harzianum* (fungus) (MI_1_) | 10 ppm (Cd_1_); 49.25 mg Cd(NO_3_)_2_ kg^-1^soil |
| Cow manure biochar (BC_2_) | *Bacillus subtilis* (bacteria) (MI_2_) | 30 ppm (Cd_2_); 147.75 mg Cd(NO_3_)_2_ kg^-1^ soil |
| Poultry manure biochar (BC_3_) | *Trichoderma harzianum* + *Bacillus subtilis* (MI_3_) |  |

**Table S3:** Analysis of variance for the influence of co-application of biochar amendment (B) and microorganisms (M) on different soil and plant traits under cadmium (Cd) stress

| Dependent variable | DF | B | Cd | M | B×Cd | B×M | Cd×M | B×Cd×M |
| --- | --- | --- | --- | --- | --- | --- | --- | --- |
| Root length | 47 | <0.001 | <0.001 | <0.001 | <0.001 | 0.0185 | <0.001 | <0.001 |
| Root fresh biomass | 47 | <0.001 | <0.001 | <0.001 | 0.0001 | <0.001 | <0.001 | 0.0310 |
| Root dry biomass | 47 | <0.001 | <0.001 | <0.001 | 0.0009 | <0.001 | <0.001 | 0.0448 |
| Shoot length | 47 | <0.001 | <0.001 | <0.001 | <0.001 | 0.0680 | <0.001 | <0.001 |
| Shoot fresh biomass | 47 | <0.001 | <0.001 | <0.001 | <0.001 | <0.001 | <0.001 | <0.001 |
| Shoot dry biomass | 47 | <0.001 | <0.001 | <0.001 | <0.001 | <0.001 | <0.001 | <0.001 |
| Soil pH | 47 | <0.001 | <0.001 | <0.001 | 0.9623 | 0.0430 | 0.9361 | 0.9328 |
| Soil electrical conductivity | 47 | <0.001 | <0.001 | <0.001 | 0.0207 | 0.1328 | 0.4927 | 0.5891 |
| Soil organic carbon | 47 | <0.001 | <0.001 | 0.0050 | 1.0000 | 0.0050 | 1.0000 | 1.0000 |
| Soil organic matter | 47 | <0.001 | <0.001 | 0.0047 | 1.0000 | 0.0047 | 1.0000 | 1.0000 |
| Total nitrogen | 47 | <0.001 | <0.001 | <0.001 | <0.001 | <0.001 | 0.0008 | <0.001 |
| Total phosphorous | 47 | <0.001 | <0.001 | <0.001 | <0.001 | <0.001 | 0.0116 | <0.001 |
| Available phosphorous | 47 | <0.001 | <0.001 | <0.001 | <0.001 | <0.001 | 0.0116 | <0.001 |
| Available potassium | 47 | <0.001 | 0.0003 | <0.001 | <0.001 | <0.001 | 0.0679 | 0.0005 |
| Electrolyte leakage | 47 | <0.001 | <0.001 | 0.0004 | <0.001 | <0.001 | 0.0001 | <0.001 |
| MDA | 47 | <0.001 | <0.001 | <0.001 | <0.001 | <0.001 | <0.001 | <0.001 |
| H_2_O_2_ | 47 | <0.001 | <0.001 | <0.001 | 1.0000 | 1.0000 | <0.001 | 1.0000 |
| SOD | 47 | <0.001 | <0.001 | <0.001 | <0.001 | <0.001 | <0.001 | <0.001 |
| CAT | 47 | <0.001 | <0.001 | <0.001 | <0.001 | <0.001 | <0.001 | <0.001 |
| POD | 47 | 0.0214 | <0.001 | <0.001 | <0.001 | <0.001 | <0.001 | <0.001 |
| Chlorophyll content | 47 | <0.001 | <0.001 | <0.001 | 0.3181 | 0.4559 | 0.1368 | 0.8208 |
| Photosynthetic rate | 47 | <0.001 | <0.001 | 0.005 | <0.001 | <0.001 | <0.001 | <0.001 |
| Transpiration rate | 47 | <0.001 | <0.001 | <0.001 | 0.2807 | 0.7957 | 0.4742 | 0.7846 |
| WUE | 47 | 0.001 | <0.001 | <0.001 | 0.0128 | 0.0006 | <0.001 | <0.001 |
| Stomatal conductance | 47 | <0.001 | <0.001 | <0.001 | 0.0105 | 0.3290 | 0.0028 | 0.0024 |
| Intercellular CO_2_ | 47 | <0.001 | <0.001 | <0.001 | <0.001 | <0.001 | <0.001 | <0.001 |
| Cd content in shoot | 47 | <0.001 | <0.001 | <0.001 | <0.001 | <0.001 | <0.001 | <0.001 |
| Cd content in root | 47 | <0.001 | <0.001 | <0.001 | <0.001 | <0.001 | <0.001 | <0.001 |
| Cd content in soil | 47 | <0.001 | <0.001 | <0.001 | <0.001 | <0.001 | <0.001 | <0.001 |

MDA = Malondialdehyde; H_2_O_2_ = Hydrogen peroxide; SOD = Superoxide dismutase; CAT = Catalase; POD = Peroxidase; WUE = Water use efficiency.

**Table S4:** Effect of co-application of biochar and microorganisms on soil pH, electrical conductivity of soil leachates, soil organic carbon, and organic matter under cadmium (Cd) contaminated soil.

| **Cd** | **MI** | **BC_0_** | **BC_1_** | **BC_2_** | | **BC_3_** | | **Mean (Cd×MI)** |
| --- | --- | --- | --- | --- | --- | --- | --- | --- |
| **Soil pH** | | | | | | | |  |
| **Cd_0_** | **MI_0_** | 8.72±0.03 a | 8.51±0.03 b-f | 8.26±0.06 j-p | | 8.45±0.11 d-h | | 8.49±0.19 A |
|  | **MI_1_** | 8.61±0.07 abc | 8.21±0.05 l-q | 8.33±0.04 g-l | | 8.29±0.04 i-n | | 8.36±0.17 CD |
|  | **MI_2_** | 8.64±0.04 ab | 8.34±0.07 g-l | 8.31±0.05 h-m | | 8.31±0.03 h-m | | 8.40±0.16 BC |
|  | **MI_3_** | 8.59±0.06 a-d | 8.34±0.05 g-l | 8.28±0.06 j-n | | 8.33±0.06 g-l | | 8.39±0.14 BC |
| **Cd_1_** | **MI_0_** | 8.65±0.10 ab | 8.36±0.17 f-k | 8.27±0.05 j-o | | 8.46±0.05 c-h | | 8.44±0.16 AB |
|  | **MI_1_** | 8.54±0.09 b-e | 8.19±0.03 l-q | 8.14±0.14 n-q | | 8.22±0.05 k-q | | 8.27±0.18 EFG |
|  | **MI_2_** | 8.52±0.09 b-e | 8.16±0.10 m-q | 8.29±0.08 i-n | | 8.25±0.06 j-p | | 8.31±0.15 DEF |
|  | **MI_3_** | 8.59±0.05 a-d | 8.30±0.05 h-m | 8.21±0.09 k-q | | 8.26±0.11 j-p | | 8.34±0.17 CDE |
| **Cd_2_** | **MI_0_** | 8.59±0.11 a-d | 8.30±0.22 h-m | 8.17±0.07 m-q | | 8.40±0.05 e-j | | 8.37±0.18 BCD |
|  | **MI_1_** | 8.44±0.13 d-i | 8.10±0.08 q | 8.11±0.21 pq | | 8.14±0.11 n-q | | 8.20±0.16 G |
|  | **MI_2_** | 8.46±0.16 c-g | 8.12±0.19 opq | 8.17±0.12 m-q | | 8.15±0.10 m-q | | 8.23±0.16 G |
|  | **MI_3_** | 8.48±0.04 c-g | 8.14±0.15 n-q | 8.19±0.07 l-q | | 8.19±0.16 l-q | | 8.25±0.16 FG |
| **Mean (BC)** | | 8.57±0.08 A | 8.26±0.12 BC | 8.23±0.07 C | | 8.29±0.11 B | |  |
| **LSD (p 0.05) value** | | Cd×MI= 0.08; BC= 0.04; Cd×MI×BC= 0.15 | | |  |  |  |  |
| **Electrical conductivity (mS cm^−1^)** | | | | | | | |  |
| **Cd_0_** | **MI_0_** | 2.87±0.30 o-s | 3.13±0.27 m-q | 4.34±0.39 c-h | | 4.03 ±0.46 e-i | | 3.59±0.70 CD |
|  | **MI_1_** | 2.76±0.23 q-u | 3.66±0.27 ijk | 5.17±0.29 a | | 4.30±0.29 c-h | | 3.97±1.02 AB |
|  | **MI_2_** | 2.98±0.29 n-s | 3.88±0.35 g-j | 5.11±0.42 a | | 4.03±0.14 e-i | | 4.00±0.87 AB |
|  | **MI_3_** | 3.16±0.18 l-q | 3.62±0.25 i-l | 5.03±0.52 ab | | 4.49±0.27 cde | | 4.07±0.84 A |
| **Cd_1_** | **MI_0_** | 2.53±0.46 s-v | 3.03±0.36 m-r | 4.24±0.24 c-h | | 3.88±0.09 g-j | | 3.42±0.78 D |
|  | **MI_1_** | 3.15±0.32 l-q | 3.44±0.46 j-n | 4.56±0.31 cd | | 3.98±0.20 f-i | | 3.78±0.62 BC |
|  | **MI_2_** | 3.08±0.28 m-r | 3.87±0.04 hij | 4.43±0.31 c-f | | 4.09±0.11 d-i | | 3.87±0.57 AB |
|  | **MI_3_** | 3.15±0.08 l-q | 3.50±0.23 j-m | 4.63±0.32 bc | | 4.35±0.25 c-g | | 3.91±0.70 AB |
| **Cd_2_** | **MI_0_** | 1.99±0.04 w | 2.13±0.16 vw | 3.25±0.25 k-p | | 2.75±0.41 q-u | | 2.53±0.58 F |
|  | **MI_1_** | 1.94±0.32 w | 2.31±0.38 uvw | 3.46±0.26 j-m | | 2.79±0.46 p-t | | 2.63±0.66 F |
|  | **MI_2_** | 2.33±0.25 t-w | 2.61±0.41 r-u | 3.70±0.24 ijk | | 3.26±0.30 k-p | | 2.98±0.62 E |
|  | **MI_3_** | 2.29±0.31 uvw | 2.29±0.32 uvw | 4.02±0.13 e-i | | 3.33±0.42 k-o | | 2.98±0.85 E |
| **Mean (B)** | | 2.69±0.46 D | 3.12±0.64 C | 4.33±0.63 A | | 3.77±0.59 B | |  |
| **LSD (p 0.05) value** | | Cd×MI= 0.24; BC= 0.14; Cd×MI×BC= 0.47 | | | | | |  |
| **Soil organic carbon (g kg^-1^)** | | | | | | | |  |
| **Cd_0_** | **MI_0_** | 3.83±0.05 n | 7.33±0.53 f-k | 9.01±0.66 abc | | 8.42±0.63 bcd | | 7.15±2.32 A |
|  | **MI_1_** | 3.67±0.36 no | 7.96±0.53 def | 9.49±0.62 a | | 8.35±0.45 cde | | 7.37±2.55 A |
|  | **MI_2_** | 3.68±0.23 no | 7.52±0.05 f-j | 9.10±0.21 ab | | 8.44±0.30 bcd | | 7.19±2.42 A |
|  | **MI_3_** | 3.66±0.20 no | 7.63±0.38 f-i | 9.50±0.49 a | | 9.09±0.11 ab | | 7.47±2.66 A |
| **Cd_1_** | **MI_0_** | 3.17±0.18 nop | 6.67±0.64 klm | 8.35±0.65 cde | | 7.76±0.61 d-h | | 6.49±2.32 BC |
|  | **MI_1_** | 3.01±0.48 op | 7.30±0.43 f-k | 8.83±0.72 abc | | 7.69±0.48 e-h | | 6.71±2.55 B |
|  | **MI_2_** | 3.02±0.38 op | 6.86±0.17 j-m | 8.44±0.26 bcd | | 7.78±0.27 d-h | | 6.52±2.42 BC |
|  | **MI_3_** | 3.00±0.14 op | 6.97±0.45 i-l | 8.83±0.53 abc | | 8.43±0.21 bcd | | 6.81±2.66 B |
| **Cd_2_** | **MI_0_** | 2.69±0.20 p | 6.19±0.67 m | 7.87±0.64 d-h | | 7.28±0.60 g-k | | 6.01±2.32 D |
|  | **MI_1_** | 2.53±0.50 p | 6.82±0.42 klm | 8.34±0.74 cde | | 7.20±0.50 h-k | | 6.22±2.55 CD |
|  | **MI_2_** | 2.54±0.41 p | 6.38±0.20 lm | 7.96±0.29 d-g | | 7.30±0.26 f-k | | 6.04±2.42 D |
|  | **MI_3_** | 2.52±0.14 p | 6.48±0.46 lm | 8.35±0.54 cde | | 7.95±0.23 d-g | | 6.32±2.66 CD |
| **Mean (BC)** | | 3.11±0.49 D | 7.01±0.54 C | 8.67±0.54 A | | 7.97±0.58 B | |  |
| **LSD (p 0.05) value** | | Cd×MI= 0.34; BC= 0.20; Cd×MI×BC= 0.68 | | | | | |  |
| **Soil organic matter (%)** | | | | | | | |  |
| **Cd_0_** | **M_0_** | 6.61±0.08 o | 12.64±0.91 g-l | 15.54±1.14 abc | | 14.52±1.08 b-e | | 12.33±4.00 A |
|  | **MI_0_** | 6.33±0.62 opq | 13.73±0.91 d-g | 16.35±1.06 a | | 14.39±0.78 c-f | | 12.70±4.39 A |
|  | **MI_1_** | 6.35±0.39 op | 12.96±0.08 g-k | 15.69±0.36 ab | | 14.55±0.52 bcd | | 12.39±4.18 A |
|  | **MI_2_** | 6.31±0.34 opq | 13.15±0.66 g-j | 16.36±0.85 a | | 15.67±0.18 ab | | 12.87±4.59 A |
| **Cd_1_** | **MI_3_** | 5.47±0.30 o-r | 11.50±1.10 lmn | 14.40±1.12 c-f | | 13.38±1.05 e-i | | 11.19±4.00 BC |
|  | **MI_0_** | 5.19±0.82 pqr | 12.59±0.74 g-l | 15.22±1.24 abc | | 13.25±0.83 f-i | | 11.56±4.39 B |
|  | **MI_1_** | 5.21±0.66 pqr | 11.82±0.29 k-n | 14.55±0.45 b-e | | 13.41±0.46 d-i | | 11.25±4.18 BC |
|  | **MI_2_** | 5.17±0.25 qr | 12.01±0.78 j-m | 15.22±0.91 abc | | 14.53±0.36 b-e | | 11.73±4.59 B |
| **Cd_2_** | **MI_3_** | 4.64±0.35 r | 10.67±1.15 n | 13.57±1.11 d-i | | 12.55±1.03 h-l | | 10.36±4.00 D |
|  | **MI_0_** | 4.36±0.86 r | 11.76±0.73 lmn | 14.39±1.27 c-f | | 12.42±0.86 i-l | | 10.73±4.39 CD |
|  | **MI_1_** | 4.38±0.71 r | 10.99±0.34 mn | 13.72±0.49 d-h | | 12.58±0.45 g-l | | 10.42±4.18 D |
|  | **MI_2_** | 4.34±0.25 r | 11.18±0.80 mn | 14.39±0.94 c-f | | 13.70±0.40 d-h | | 10.90±4.59 CD |
| **Mean (BC)** | | 5.36±0.85 D | 12.08±0.94 C | 14.95±0.93 A | | 13.75±1.00 B | |  |
| **LSD (p 0.05) value** | | Cd×MI= 0.59; BC= 0.34; Cd×MI×BC= 1.18 | | | | | |  |

Values are representing means ±standard deviation (SD) of three replication. Means within a column sharing the same letters do not differ significantly different at *P*≤ 0.05. BC= biochar types; Cd= artificial cadmium stress; MI= microbial inoculation; BC_0_= zero biochar; BC_1_= maize-straw synthesized biochar, BC_2_= cow-manure synthesized biochar; BC_3_= poultry-manure synthesized biochar; MI_0_ = no microbial inoculation; MI_1_= *Trichoderma harzianum* (fungus) microbial inoculation; MI_2_= *Bacillus subtilis* (bacteria) microbial inoculation; MI_3_= combined *Trichoderma harzianum* and *Bacillus subtilis* microbial inoculation; Cd_0_= 0 ppm Cd; Cd_1_= 10 ppm Cd; Cd_2_= 30 ppm Cd.


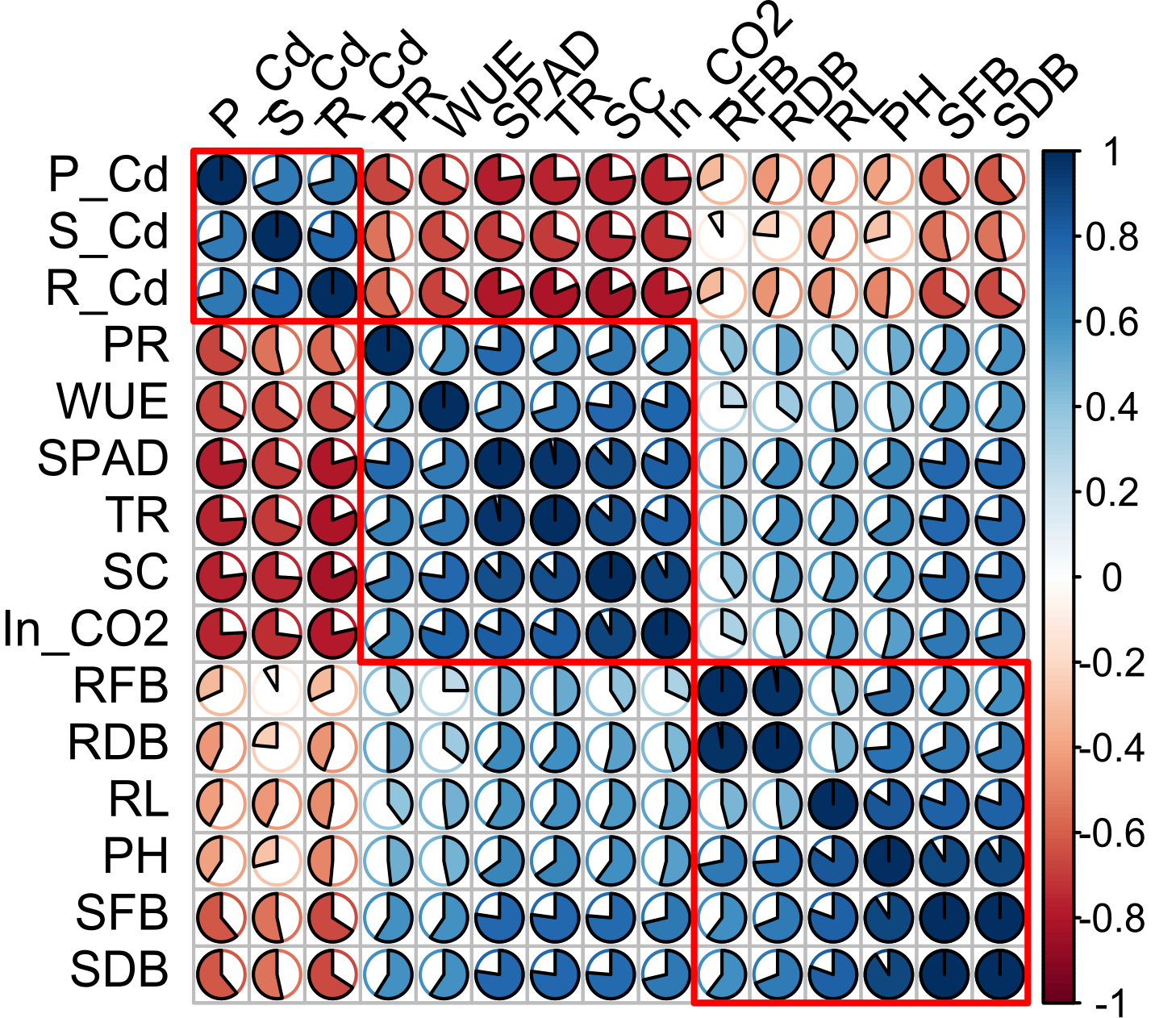


**Fig.** **S1:** Pearson’s correlation among various traits under Cd stress in maize.

P_Cd= cadmium concentration in plant shoot; cadmium; S_Cd= cadmium concentration in soil; R_Cd= cadmium concentration in plant root; PR= photosynthesis rate; WUE= water use efficiency; SPAD= chlorophyll SPAD value; TR= transpiration rate; SC= stomatal conductance; In CO_2_= Intercellular carbon dioxide; RFB= root fresh biomass; RDB= root dry biomass; RL= root length; PH= plant shoot height; SFB= plant shoot fresh biomass; SDB= plant shoot dry biomass.
